# Medulloblastoma cerebrospinal fluid reveals metabolites and lipids indicative of hypoxia and cancer-specific RNAs

**DOI:** 10.1186/s40478-022-01326-7

**Published:** 2022-02-24

**Authors:** Bongyong Lee, Iqbal Mohamad, Rudramani Pokhrel, Rabi Murad, Menglang Yuan, Stacie Stapleton, Chetan Bettegowda, George Jallo, Charles G. Eberhart, Timothy Garrett, Ranjan J. Perera

**Affiliations:** 1 Department of Oncology, Sidney Kimmel Comprehensive Cancer Center, School of Medicine, Johns Hopkins University, 1650 Orleans St, Baltimore, MD 21231 USA; 2 Johns Hopkins All Children’s Hospital, 600 5th St. South, St. Petersburg, FL 33701 USA; 3 Department Pathology, Immunology and Laboratory Medicine, College of Medicine, University of Florida, 1395 Center Drive, Gainesville, FL 32610 USA; 4 Sanford Burnham Prebys Medical Discovery Institute, 10901 N. Torrey Pines Road, La Jolla, CA 92037 USA; 5 Department of Neurosurgery, Johns Hopkins University School of Medicine, Baltimore, USA; 6 Department of Pathology, Johns Hopkins University School of Medicine, 720 Rutland Avenue, Baltimore, MD 21205 USA; 7 Department of Bioinformatics and Computational Biology, The University of Texas MD Anderson Cancer Center, Houston, TX 77030 USA

**Keywords:** Circular RNA, Lipidomics, Medulloblastoma, Metabolomics, TCA, Transcriptomics

## Abstract

Medulloblastoma (MB) is the most common malignant brain tumor in children. There remains an unmet need for diagnostics to sensitively detect the disease, particularly recurrences. Cerebrospinal fluid (CSF) provides a window into the central nervous system, and liquid biopsy of CSF could provide a relatively non-invasive means for disease diagnosis. There has yet to be an integrated analysis of the transcriptomic, metabolomic, and lipidomic changes occurring in the CSF of children with MB. CSF samples from patients with (n = 40) or without (n = 11; no cancer) MB were subjected to RNA-sequencing and high-resolution mass spectrometry to identify RNA, metabolite, and lipid profiles. Differentially expressed transcripts, metabolites, and lipids were identified and their biological significance assessed by pathway analysis. The DIABLO multivariate analysis package (R package mixOmics) was used to integrate the molecular changes characterizing the CSF of MB patients. Differentially expressed transcripts, metabolites, and lipids in CSF were discriminatory for the presence of MB but not the exact molecular subtype. One hundred and ten genes and ten circular RNAs were differentially expressed in MB CSF compared with normal, representing TGF-β signaling, TNF-α signaling via NF-kB, and adipogenesis pathways. Tricarboxylic acid cycle and other metabolites (malate, fumarate, succinate, α-ketoglutarate, hydroxypyruvate, N-acetyl-aspartate) and total triacylglycerols were significantly upregulated in MB CSF compared with normal CSF. Although separating MBs into subgroups using transcriptomic, metabolomic, and lipid signatures in CSF was challenging, we were able to identify a group of omics signatures that could separate cancer from normal CSF. Metabolic and lipidomic profiles both contained indicators of tumor hypoxia. Our approach provides several candidate signatures that deserve further validation, including the novel circular RNA circ_463, and insights into the impact of MB on the CSF microenvironment.

**Supplementary Information:**

The online version contains supplementary material available at 10.1186/s40478-022-01326-7.

## Introduction

Medulloblastoma (MB) is the most common malignant tumor of the cerebellum in children, and it accounts for 10–15% of pediatric central nervous system (CNS) tumors [1]. MB has a propensity to invade and disseminate in the cerebrospinal fluid (CSF), with disseminated CNS disease occurring in 30–40% of cases at initial diagnosis and most patients at recurrence [2]. The current diagnosis of MB is based on clinical assessment, imaging, and subsequent histopathological examination of biopsies, with magnetic resonance imaging (MRI) and lumbar puncture often performed to monitor treatment responses and to detect recurrences [3]. Although recent advances in imaging have improved MB detection and monitoring, there remain unmet needs for diagnostics to sensitively detect the disease at both initial presentation and at recurrence. This latter need is particularly important, since recurrences (particularly subependymal metastatic disease within the ventricles) do not always enhance on MRI, and, when present, herald incurable disease that is nearly always fatal [4, 5].

The 2016 World Health Organization Classification of Tumors of the Nervous System reclassified MB into four subtypes: WNT (wingless) activated, SHH (sonic hedgehog) activated, group 3, and group 4 based on histopathological and molecular features [6]. More recent studies with increased cohort sizes have identified intra-subtypes and described a total of twelve subgroups [7, 8]. Despite this considerable progress in the molecular characterization of MB, the biology and impact of the disease on the CSF microenvironment is still poorly understood, despite the tumor microenvironment contributing to cancer progression, metastasis, and resistance and potentially providing a rich source of biomarkers that can be sampled relatively non-invasively to chart the course of disease.

Liquid biopsies—the molecular analysis of biofluids—is a minimally-invasive method that shows promise for disease detection and monitoring through the measurement of circulating tumor cells, DNA, RNA, or extracellular vesicles in the urine, CSF, and blood samples [9]. Although blood has most commonly been used as the biofluid of choice for liquid biopsy, its sensitivity for CNS tumors tends to be poor due to biomarkers of interest not crossing the blood–brain barrier [10]. However, CSF bathes the brain and spinal cord and therefore provides a window to tumors arising in the CNS and disseminating in the CSF. Furthermore, many patients with MB have hydrocephalus that needs to be drained to reduce intracranial pressure and prior to surgery. Many studies have attempted to detect biomarkers in the CSF in adult patients with CNS tumors [11], but few have analyzed the metabolite, lipid, transcriptomic, and genomic profiles in the CSF of children [10, 12–15]. To date, there has yet to be an integrated analysis of the transcriptomic, metabolomic, and lipidomic changes occurring in the CSF of children with MB. This is in no small part due to technical difficulties in: (i) global RNA-sequencing of messenger RNAs (mRNAs) and circular RNAs (circRNAs) in CSF, which contains low concentrations of RNAs that are susceptible to fragmentation and degradation; and (ii) the ability to profile metabolites and lipids, which have only recently been facilitated by the advent of high-resolution, high-sensitivity, and high mass accuracy mass spectrometers [16].

To obtain an integrated understanding of the pathobiological impact of MB on the surrounding microenvironment of the CSF and as a precursor to biomarker identification, we analyzed the transcriptomic, metabolomic, and lipidomic landscapes of CSF samples obtained from forty patients with primary or recurrent MB and eleven normal controls. In doing so, we establish that patients with MB have a unique transcriptomic, metabolomic, and lipidomic landscape in their CSF that might be helpful for diagnosis and monitoring and that reflects biological changes consistent with the presence of MB in the CNS.

## Materials and methods

### CSF samples

Details of the CSF samples analyzed are shown in Additional file 4: Table S1. The Institutional Review Board (IRB) at each institution approved the protocol for CSF collection, and all patients provided written informed consent. The eleven normal samples were purchased from BioIVT (Westbury, NY USA), Discovery Life Sciences (Huntsville, AL USA), and Lee Biosolutions (Maryland Heights, MO USA); thirty samples were from the Children Brain Tumor Tissue Consortium (CBTTC); five samples were from Johns Hopkins University (JHU); and five samples from Johns Hopkins All Children's Hospital (JHACH). Cell-free CSF samples were snap-frozen without further processing and stored at − 80 °C until sample preparation.

### Total RNA isolation from CSF and library preparation for RNA-seq

Briefly, 0.2 ml of CSF was mixed with 1 ml of QIAzol (Qiagen, Hilden, Germany) and incubated for 5 min at room temperature. Next, 0.4 ml of chloroform was added and mixed. The aqueous phase was obtained by centrifugation at 14,000 × *g* for 15 min at 4 °C, and RNAs were isolated using the miRNeasy Mini kit (Qiagen) according to the manufacturer’s protocol. To perform library generation with the NuGen Ovation Solo system, the purified RNAs were concentrated using the RNA Clean & Concentrator kit (Zymo Research Corp), and libraries were prepared according to the manufacturer's instructions. Library quantities were estimated using a KAPA library quantification kit (Roche Sequencing and Life Science, Wilmington, MA).

### cDNA generation and whole transcriptome amplification from CSF for quantitative real-time PCR (qRT-PCR) validation

Total RNAs were isolated from 0.1 ml CSF using a miRNeasy Mini kit (Qiagen) and further concentrated using the RNA Clean & Concentrator kit (Zymo Research Corp). cDNA generation and whole transcriptome amplification were performed using a REPLI-g WTA single cell kit (Qiagen) according to the manufacturer's instructions. 10 ng of amplified cDNA was used for the qRT-PCR reaction. qRT-PCR was performed using a Power SYBR Green PCR master mix (Applied Biosystems, Waltham, MA) in the QuantStudio 3 and 5 Real-Time PCR Systems (Thermo Fisher Scientific, Waltham, MA) as previously described [17]. The average Ct value of two genes, beta-actin (*ACTB*) and ribosomal protein S28 (*RPS28*), were used as endogenous controls. The primer sequences for the genes are listed in Additional file 3.

### Global metabolite and lipid extraction

For global metabolomics, 50 µL of CSF samples thawed on ice were used. For monitoring metabolite extraction, 20 µL of metabolite internal standard mixture containing L-leucine-D10 (4 µg/mL), L-tryptophan-2,3,3-D3 (40 µg/mL), (4 µg/mL), L-tyrosine-13C6 (4 µg/mL), caffeine-D3 (4 µg/mL), succinic acid-2,3,3,3-D4 (4 µg/mL), L-leucine-13C6 (4 µg/mL), L-phenylalanine-13C6 (4 µg/mL), N-BOC-L-tert-leucine (4 µg/mL), and N-BOC-L-aspartic acid (4 µg/mL) in 0.1% formic acid in water was added to CSF prior to protein precipitation. L-leucine-D10, creatine-D3, L-tryptophan-2,3,3-D3, succinic acid-2,3,3,3-D4, and caffeine-D3 were purchased from CDN Isotope (Pointe-Clarie, Quebec, Canada). L-tyrosine-13C6, L-leucine-13C6, and L-phenylalanine-13C6 were purchased from Cambridge Isotope laboratories, Inc. (Tewksbury, MA). N-BOC-L-tert-leucine and N-BOC-L-aspartic acid were purchased from Acros Organics (Fair Lawn, NJ). After adding the internal standard mixture, the samples were vortex mixed and stored on ice.

Global metabolite extraction was performed using 1 mL ice-cold methanol (80%) for 20–30 min with occasional vortexing. The samples were centrifuged at 20,000 rpm for 10 min at 4 °C to pellet. The supernatant (500 µL) was transferred to a new tube and dried under nitrogen gas flow at 30 °C. The dried sample was reconstituted in 0.1% formic acid in water (50 µL) containing injection standards including BOC-L-tyrosine (2 µg/mL), BOC-L-tryptophan (2 µg/mL), and BOC-D-phenylalanine (2 µg/mL). The remaining 500 µL of supernatant from methanol precipitation was transferred to 15 mL glass tubes for global lipidomic extraction following a modified version of the Folch extraction [18]. Briefly, 20 μL of internal standard mixture containing lysophosphatidylcholine (LPC 17:0), phosphatidylserine (PS 14:0/14:0), phosphatidylcholine (PC 17:0/17:0), phosphatidylglycerol (PG 14:0/14:0), phosphatidylethanolamine (PE 15:0/15:0), sphingomyelin (SM d18:1/17:0), ceramide (Cer d18:1/17:0), diacylglycerol (DG 14:0/14:0), triacylglycerol (TG 15:0/15:0/15:0), bis(monacyl-glycero)phosphate (BMP 14:0 (S,R), and Lyso SM(d17:1) each at 100 ppm in 2:1 chloroform:methanol was added. Except for TG, all other lipid standards were purchased from Avanti Polar Lipids (Alabaster, AL), while TG was purchased from Sigma-Aldrich (St. Louis, MO). Extraction was performed by adding ice cold 4:2:1 chloroform:methanol:water (v:v:v), and the organic phase was collected using low speed centrifugation at 3500 rpm for 10 min at 4 °C. Collected organic phase was dried down under nitrogen flow and reconstituted in 50 μL of isopropanol plus 1 μL of injection standard mixture containing LPC(19:0), PC(19:0/19:0), PG(17:0/17:0), PE(17:0/17:0), PS(17:0/17:0), and TG(17:0/17:0/17:0) each at 100 ppm in 2:1 chloroform:methanol. Metabolomic and lipidomic samples were run separately and, for each sequence, solvent blanks, extraction blanks (without internal standard), neat quality controls (amino acid internal standards mixtures), pooled samples (normal pooled mixture of all normal replicates, 5 µL each and cancer pooled mixture of all cancerous samples, 5 µL each) were also prepared for evaluation of extraction and data collection efficiency.

### Metabolomic data acquisition

High-pressure liquid chromatography coupled to high-resolution tandem mass spectrometry (LC-HRMS/MS) was used for data collection. Chromatographic separation for metabolomics was achieved using reversed phase chromatography with a C18-pfp column (Ace, Aberdeen, Scotland; 100 × 2.1 mm, 2 µm). The mobile phases consisted of solvent A (0.1% FA in H_2_O) and solvent B (acetonitrile). The system was held constant from 0–3 min at 100% A, then mobile phase B was ramped from 0 B to 80% over 10.0 min (3–13 min) and then held constant at 80% B for 3 min (13–16 min) with a flow rate of 350 µL/min and column temperature of 25 °C. For equilibration, the system was returned to initial conditions with 0% B and the flow rate was increased to 600 µL/min. The flow rate was reduced back to 350 µL/min before the next injection. The data collection time per sample was 20.50 min. Both positive (injection volume 2 µL) and negative ion polarity (injection volume 3 µL) in full scan mode (35,000 mass resolution) were acquired.

### Lipidomic data acquisition

Chromatographic separation for lipidomics was achieved on a Waters Acquity C18 BEH column maintained at 50 °C (2.1 × 100 mm, 1.7 μm particle size, Waters, Milford, MA). The mobile phases consisted of solvent A (60:40 acetonitrile:water) and solvent B (90:8:2 isopropanol:acetonitrile:water), both with 10 mM ammonium formate and 0.1% formic acid. The gradient elution was ramped from 20% D to 98% D with a 0.5 mL/min flow rate over 17.00 min followed by 3.00 min column flush and re-equilibration. The flow rate was 500 μL/min. Samples were analyzed in positive and negative electrospray ionization on a Thermo Scientific Q-Exactive mass spectrometry with Dionex Ultimate 3000 UHPLC (Thermo Scientific, San Jose, CA). Data-dependent (ddMS2-top5) MS/MS and AIF (All-ion fragmentation) data were obtained on pooled samples per group for identification purposes.

### Tumor vs normal total RNA analysis

We used the ultra-fast FASTQ preprocessor package fastp [19] for quality control and filtering the fastq read data of CSF samples. STAR 2.7 [20] was used to aligned the filtered fastq files to Ensemble human genome v100. The read counts form aligned bam files were quantified using the featureCounts package [21]. One normal CSF sample was removed from downstream analysis, since it had an extremely low gene count. Additionally, low count genes from raw data (total expression across the sample < 2) were removed. The count data were then normalized using trimmed mean of M-values (TMM) scale normalization using edgeR [22]. Those genes with counts per million reads mapped (CPM) values > 2 in at least in three samples were chosen for downstream analysis. We used the limma-voom [23] workflow to identify the differentially expressed (DE) genes and gene signatures for two groups: MB vs normal. Heatmaps and volcano plots were plotted using R version 4.0.3.

### Tumor vs normal circular RNA analysis

To remove the ribosomal RNA (rRNA) from reads, fastq files were first mapped to human ribosomal DNA complete repeating unit (GenBank: U13369.1) using bowtie-2 read aligner [24]. The unmapped reads were filtered and extracted using a combination of samtools and bedtools for circular RNA detection. The human reference DNA and gene annotation files were downloaded from Ensembl v100. The reads were aligned to the human reference genome to generate SAM files using the BWA-MEM tool. The CIRI2 [25] work-flow was used for circular RNA (circRNA) detection from aligned SAM files. The circRNAs identified by CIRI2 were aggregated to an RNA vs sample count matrix format using the circM tool [26]. Sample CBTTC-3459 was removed since it had an extreme circRNA count compared with other samples for differential analysis. Analysis of differentially expressed circRNA was performed with the DEseq2 R package [27]. circRNA counts were very small compared with total RNA counts, so we preferred DEseq2 to limma to increase the sensitivity of differential analysis. The p-values were adjusted using the Benjamini & Hochberg method for controlling the false discovery rate. Python and R packages were used to generate plots and graphs for circRNA expression.

### Tumor vs normal global metabolite and lipid processing and identification

For lipidomics data analysis, LipidMatch Flow was used for file conversion, peak picking (implementing MZMine 2 [28]), blank filtration, lipid annotation [29], and combining positive and negative datasets. LipidMatch Flow was used to annotate ions using data-dependent MS/MS analysis. For metabolomics data analysis, metabolites were identified with MZmine 2.0 and matching metabolite retention time and *m/z* values to an internal library of over 1000 metabolites representing level 1 identification following metabolomics standards initiative guidelines. MetaboAnalyst 5.0 [30] was used for data processing with the following parameters: peak intensity table, samples in columns unpaired, missing value estimation used to replace by a small value (half of the minimum positive value in the original data, none of the features were removed in this step), data filtering by relative standard deviation (RSd = SD/mean), normalized by sum (to correct the instrumental and the technical variation), data transformed using log transformation, and data scaled using autoscaled (to allow a more direct comparison between features of greatly varying intensities). Principal component analysis (PCA), an unsupervised statistical model, and hierarchical clustering heatmap analysis were employed to visualize variance and emphasize variations in both metabolomic and lipidomic analyses. Metabolic pathway analysis was conducted using the Kyoto Encyclopedia of Genes and Genomes (KEGG) pathway database by matching metabolite sets with human metabolome (https://www.genome.jp/kegg/pathway.html). Metabolite set enrichment (fold enrichment) was investigated using MetaboAnalyst (open source R package).

### mixOmics data integration

Diablo models from mixOmics R package [31] were used to perform integrative analysis of transcriptomics, metabolomics, and lipidomics data. Thirty patient CSF samples (6 normal, 24 cancer) with all three omics datasets were taken for integration. The output of each dataset from their analyses described above was sorted according to p-values, and the top 100 features from each dataset were input into DIABLO for analysis.

## Results and discussion

### Transcriptomic profiles of CSF from patients with and without medulloblastoma

Most studies attempting to profile CSF have focused on circulating tumor DNA (ctDNA) due to the relative ease of analysis of stable DNA fragments, including in MB [12, 32, 33]. Despite CSF also containing RNAs, due to their low abundance and lability, most studies have used targeted approaches to profile miRNAs and mRNAs in CSF from patients with various CNS tumors. Recognizing the need to systematically profile RNAs in biofluids due to their biomarker potential, Hulstaert et al. recently published a comprehensive atlas of the extracellular transcriptomes of human biofluids, including CSF, but their analysis was limited to a comparison of profiles of patients with hydrocephalus and glioblastoma and no MB patient was profiled [34]. There has yet to be a comprehensive and systematic analysis of RNA species in the CSF of MB patients.

**Fig. 1:**
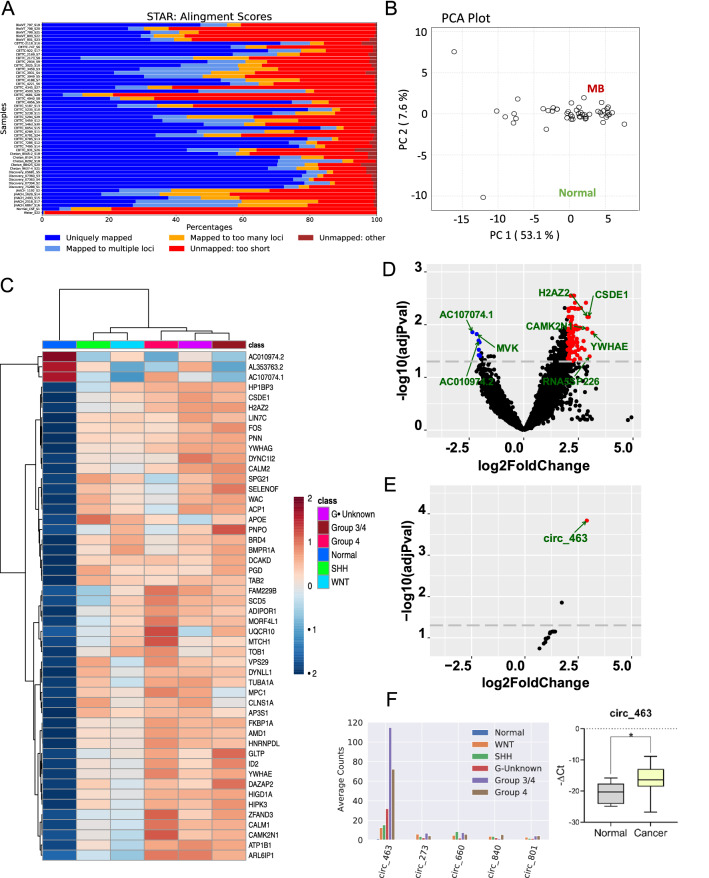
Global transcriptomic differences in the CSF of patients with (n = 40) and without (n = 11) MB. **A** Mapping rate of each CSF sample. **B** Principal component analysis of CSF samples using the 48 most differentially expressed genes showing clear separation of normal CSF samples from MB CSF samples. **C** Unsupervised clustering of samples using the 48 most differentially expressed genes showing clear separation of normal CSF samples from MB CSF samples. **D** Volcano plot showing significantly up- or downregulated genes in CSF. **E** Volcano plot for differentially expressed circRNAs between normal vs MB CSF samples. **F** Top 5 circRNAs expression in different subgroups and qRT-PCR validation of circ-463

We therefore established global transcriptomic differences in the CSF of patients with (n = 40) and without (n = 11) MB representing different molecular subtypes (Additional file 4: Table S1). Each CSF samples showed varied read counts and mapping rate (Fig. 1a). By both principal component analysis (PCA) and unsupervised clustering, CSF samples separated into two distinct groups according to the presence or absence of MB (Fig. 1b–d). Although, there was no clear separation into molecular subtypes, one hundred and ten genes were differentially expressed in CSF samples from patients with and without MB (Fig. 1c, Additional file 4: Table S2, and Additional file 4: Fig. S1; log2 fold-change (FC) < -2 or > 2; adjusted p-value < 0.05) that were enriched for several pathways by geneset enrichment analysis (GSEA) [35]: TGF-β signaling (*SKI, FKBP1A, ID2, RHOA, BMPR1A*; false discovery rate (FDR) 2.59E-04), TNF-α signaling via NF-kB (*TSC22D1, DUSP1, ID2, KLF9, FOS, IL6ST, SAT1*; FDR 1.19E-03), and adipogenesis (*ALDH2, CMPK1, APOE, UQCR10, TOB1, YWHAG*; FDR 4.51E-03). TGF-β has previously been implicated in the progression of MB [36], perhaps by suppressing the anti-tumor effects of cytotoxic T cells [37], and the other identified pathways warrant further exploration.

We next examined expression of circular RNAs (circRNAs), a novel class of non-coding (nc)RNAs with a covalently closed loop structure derived from the host gene’s RNA splicing by back splicing. Although generally present at low abundance [38], since circRNAs do not have exposed ends, they are more resistant to degradation and more stable than linear RNAs [39], making them ideal biomarkers for detection in human biofluids including blood [40], saliva [41], semen [42], urine [43], and CSF [34]. CircRNA expression levels in CSF were low, ranging from mean read counts 203 to 1850 in samples from MB patients and only 8.57 ± 5.09 in normal samples. Nevertheless, 10 circRNAs were differentially expressed between MB and non-MB groups (log2 FC < -1 or > 1; adjusted p-value < 0.1) (Fig. 1e, Additional file 4: Table S3, Additional file 1). Of these, circ_463 was the most upregulated and abundant circRNA in MB CSF, as confirmed by qRT-PCR (Fig. 1f).

Circ_463, also known as ciRS-7 or CDR1as, was originally identified as a highly expressed circRNA in human and mouse brains [44]. It contains 73 miR-7 seed targets and functions as a miR-7 sponge with an unknown role in the brain [45]. In cancers, ciRS-7 promotes growth and metastasis of esophageal squamous cell carcinoma [46], and its silencing in melanoma drives IGF2BP3-mediated invasion and metastasis [47]. In multiple myeloma, its expression is downregulated in immunomodulatory drug resistant cell lines, and depletion of ciRS-7 increased the CpG methylation of its host gene *LINC00632* [48]. While there have been a few very recent reports of circRNA expression in MB tissues and cells demonstrating potential oncogenic function for overexpressed transcripts [49–51], this is the first circRNA analysis of CSF in MB patients. Therefore, circ_463 appears to be pleiotropic, with overexpression in CSF samples of MB patients suggesting a novel oncogenic role in this context.

### The metabolic differences in CSF from patients with and without medulloblastoma

Global metabolomics has become an important unbiased approach to identify diagnostic, prognostic, and predictive biomarkers in human disease [17, 52], and altered metabolism is a hallmark of cancer cells, which need to adapt to their nutrient-poor microenvironment to sustain their viability [53]. Although it is clear that cancer cells have altered metabolism, it is less clear to what extent this influences the CNS microenvironment and the CSF. Like other tumors, several studies have established that metabolism is altered in primary and recurrent MB, including decreased fatty acid oxidation, increased lipogenesis, and a glycolytic phenotype reflected in the detection of MB by ^18^FDG-PET [54]. However, there have been fewer comprehensive studies of the CSF metabolome in CNS tumors and in MB specifically. Metabolite analysis of the CSF in glioma patients identified differences in the abundance of 43 metabolites compared with controls [55], while in MB, Reichl et al. detected upregulation of hypoxia-induced proteins and metabolites (up-regulation of tryptophan, methionine, serine and lysine) in MB CSF [56]. However, the full metabolomic landscape of CSF in MB has not been accurately or fully quantified.

**Fig. 2:**
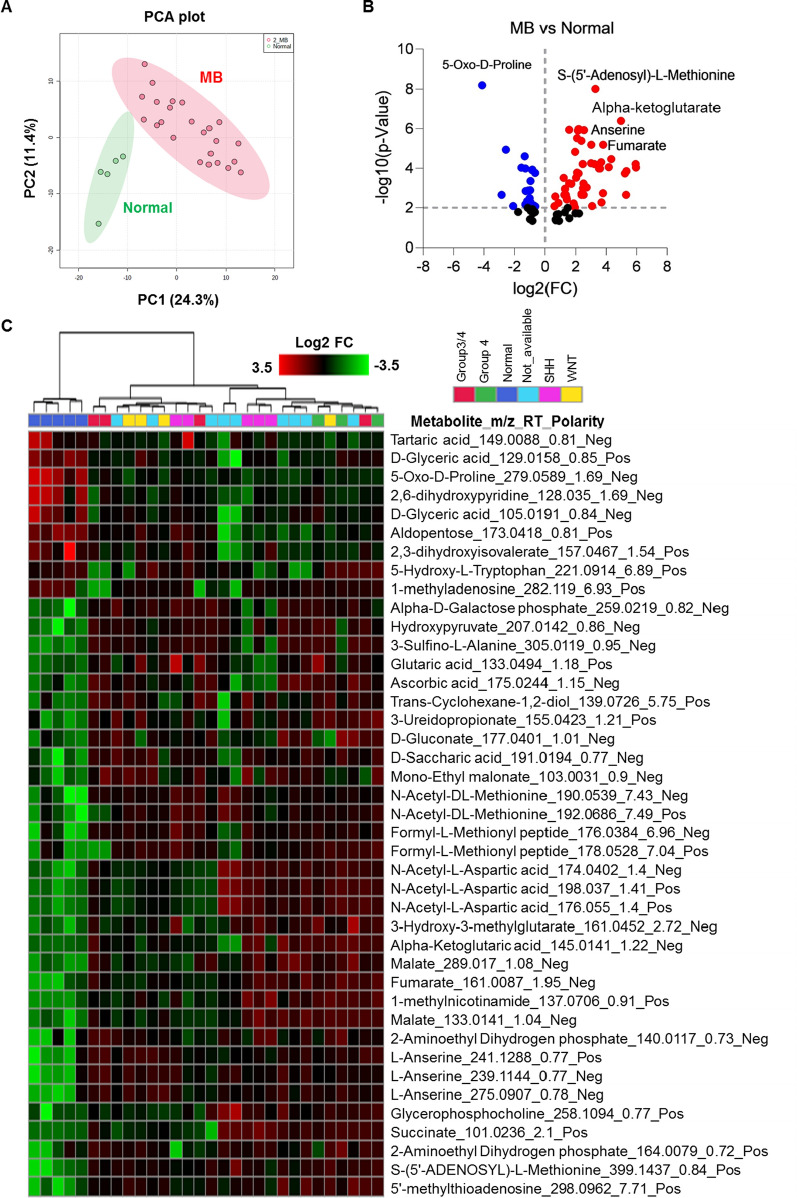
Global metabolic alterations in MB. **A** Unsupervised PCA-based multivariate analysis of CSF global metabolic profile between normal (n = 6) and MB (n = 28). **B** Volcano plot of differentially altered metabolites in MB compared with normal. **C** Relative abundance heatmap of highly altered metabolites in normal and different MBs, shown as a heatmap representation. Log2 FC, log2 fold-change; m/z, mass charge ratio; RT, retention time

Therefore, we performed comprehensive untargeted metabolic profiling of the brain CSF samples using ultra high-pressure liquid chromatography and high-resolution mass spectrometry (UHPLC-HRMS). Metabolite data were collected in a randomized manner to avoid bias. Using flank feature filtering (BFF) to eliminate false peaks, 3995 true metabolic features were identified, of which 352 metabolites were identified as level 1 (highest level of confidence in the annotation). Similar to the transcriptomic profiles, PCA and unsupervised clustering of differentially expressed metabolites revealed clear separation of metabolic profiles between normal and MB CSF (Fig. 2a and c) but not between different molecular subtypes. The majority of differentially regulated metabolites (FC > 1.5; FDR p < 0.05) were upregulated in MB samples (Fig. 2b and Additional file 4: Table S4). Exploratory pair-wise metabolite profile discrimination between normal and different MB sub-groups confirmed that differentially expressed metabolites clearly distinguished different molecular subgroups of MB (Additional file 4: Fig. S2). Uniquely elevated (Additional file 4: Fig. S3) and downregulated (Additional file 4: Fig. S4) metabolites in the different MB subtypes were analyzed using volcano plot-based differential statistical analysis (p-value < 0.05, fold change ≥ 1.5).

**Fig. 3:**
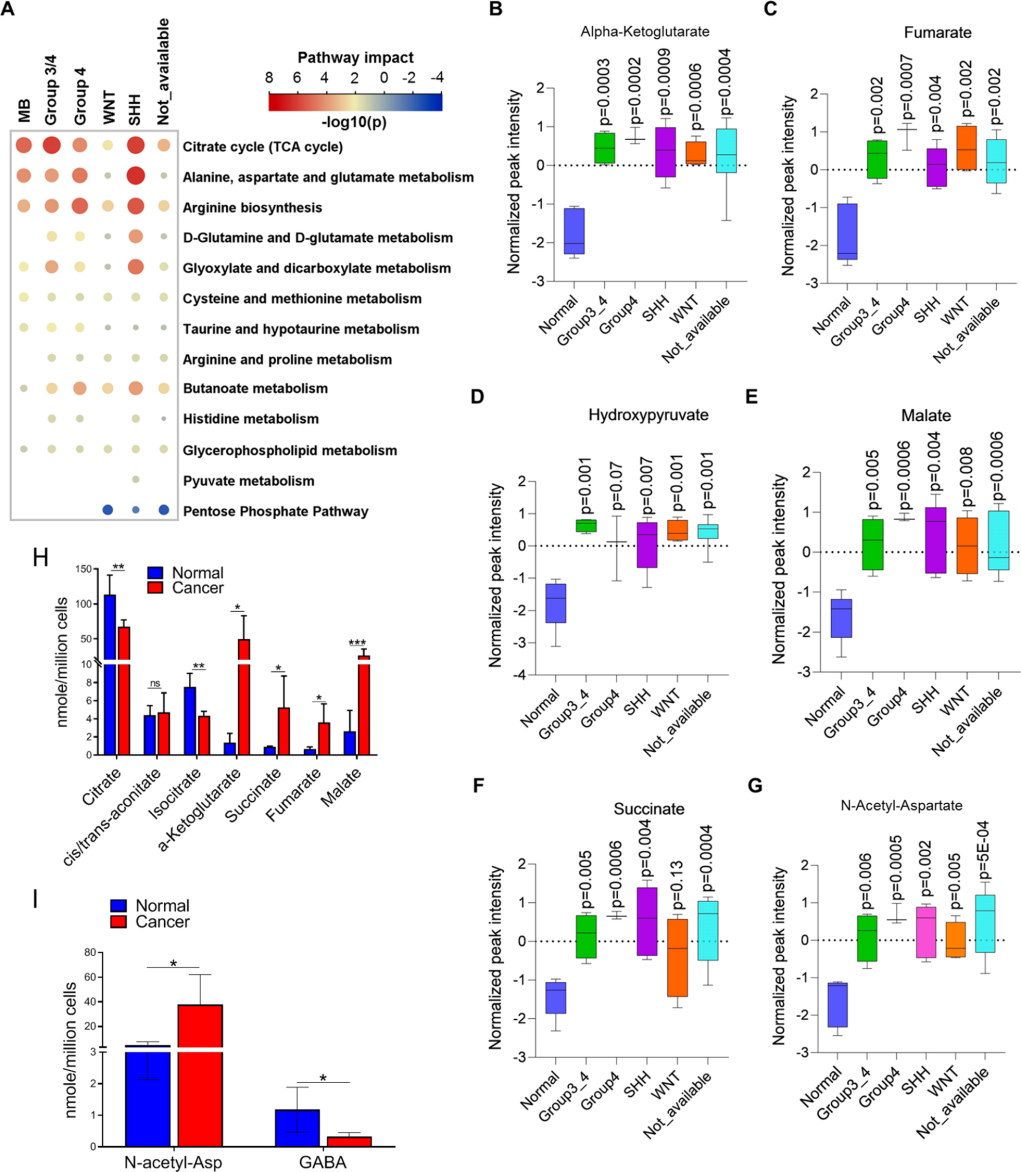
Top enriched metabolic networks in MB. **A** Enriched KEGG pathways in CSF metabolites in MB are shown with the p-values and the number of metabolites represented in each pathway. The size of each bubble represents the number of metabolites differentially expressed for each pathway. **B**-**G** Relative abundance of highly altered metabolites involved in TCA cycle and alanine, aspartate, and glutamate metabolism from untargeted metabolomics analysis. **H**, **I** Concentrations of TCA cycle metabolites **H** and N-acetyl-aspartate **I** in MB CSF compared with normal using a targeted quantitative assay

We next performed KEGG metabolic pathway analysis of significantly differentially expressed metabolites (hypergeometric test, relative betweenness centrality, p-value < 0.05) (Fig. 3a). The TCA cycle, alanine, aspartate, and glutamate metabolism, and arginine biosynthesis pathways were all upregulated in MB, particularly in SHH, group 3/4, and group 4 tumors. Given that CSF metabolic profiles did not discriminate between molecular subgroups, we established which metabolites were uniformly expressed in all MB subtypes and might therefore be candidate diagnostic biomarkers for MB. α-ketoglutarate (Fig. 3b), fumarate (Fig. 3c), hydroxypyruvate (Fig. 3d), malate (Fig. 3e), and succinate (Fig. 3f) from the TCA cycle and N-acetyl-aspartate (Fig. 3g) from the alanine, aspartate, and glutamate metabolism pathway were all significantly elevated in all different sub-groups of MB; citrate, isocitrate, and trans-aconitate (Additional file 4: Fig. S5A-C; TCA cycle) and GABA (Additional file 4: Fig. S5D; alanine, aspartate, and glutamate metabolism) showed minor but significant downregulation in MB. For validation, α-ketoglutarate, fumarate, malate, and succinate (Fig. 3h) from the TCA cycle and N-acetyl-aspartate were all significantly upregulated by targeted quantification (Fig. 3i). Finally, anserine (Additional file 4: Fig. S5E; histidine and beta-alanine metabolism) and S-(5′-adenosyl)-L-methionine (arginine biosynthesis; Additional file 4: Fig. S5F) were significantly upregulated and 5-oxo-L-proline (glutamine and glutamate metabolism; Additional file 4: Fig. S5G) significantly downregulated in MB compared with normal. Collectively, these data suggest that a broad range of metabolites in the CSF, particularly those involved in the TCA cycle, distinguish MB from normal. This is consistent with a more general model of proliferating MB cells not only using the TCA cycle to fuel the need for reducing equivalents in the form of NADPH [53] but to provide metabolic precursors for the biosynthesis on non-essential amino acids, since upregulated α-ketoglutarate indicates (i) a continuous supply of glutamine maintaining the integrity of the cell cycle [57]; (ii) maintaining the cell’s ability to synthesize citrate for energy production and de novo lipogenesis, since α-ketoglutarate is oxidized to oxaloacetate to maintain citrate production and oxaloacetate can be converted to malate and then pyruvate to produce NADPH in a glucose-independent manner [58].

### Lipidomic alterations in medulloblastoma CSF

Lipids are fundamental and abundant biomolecules in cells that have structural, transport, energy storage, and cellular signaling roles. Unsurprisingly, therefore, they all play critical roles in many diseases including cancer [59]; however, there is little available information on the lipid profiles of human MB. Tissue analysis suggests that human MBs may have high lipid levels, at least in contrast to other pediatric brain tumors [60], and a lipidomic analysis of a mouse model of SHH MB determined 34 upregulated lipids associated with metastasis [61]. Given that biofluid lipidomes might provide a rich source of biomarkers and provide insights into the underlying biology of MB, we proceeded to examine CSF lipid profiles.

**Fig. 4:**
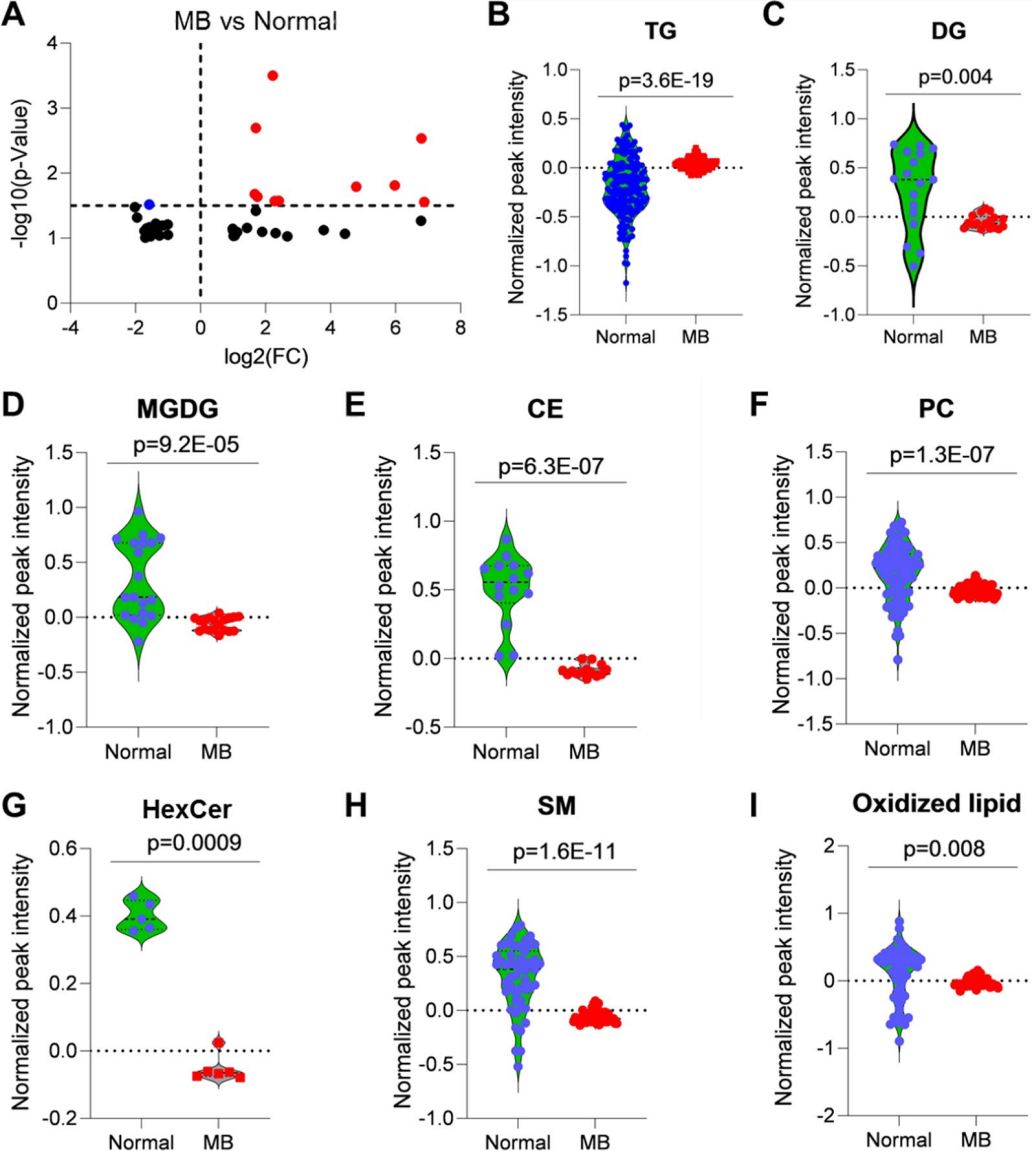
Global lipid alterations in MB from lipidomics. **A** Differentially altered lipids in MB compared with normal by volcano plot analysis. **B**-**I** Relative abundance of different lipids analyzed based on lipid class. p-values calculated based of Student’s *t*-test and two-tailed unequal variance

Using LipidMatch, 727 lipids were identified in all samples including predicted lipids (Additional file 2), and 14 of these were differentially expressed based on fold change threshold 1.5 and p-value < 0.05 (11 lipid species elevated and 3 downregulated) in the CSF of MB patients compared with normal (Fig. 4a). To understand the role of specific lipids in MB, we conducted lipid class analysis between MB and normal. Total triacylglycerols (TGs; n = 171) were significantly upregulated in MB (Fig. 4b) and diacylglycerols (DGs; n = 17) (Fig. 4c), monogalactosyldiacylglycerol (MGDG; n = 19) (Fig. 4d), cholesterol ester (CE; n = 14) (Fig. 4e), phosphatidylcholine (PC; n = 85) (Fig. 4f), N-hexadecanoyl hexosylceramide (HexCer; n = 6) (Fig. 4g), sphingomyelin (SM; n = 51) (Fig. 4h), and oxidized lipids including oxLPC (lysophosphatidylcholine; n = 2), oxLPE (lysophosphatidylethanolamine n = 1), oxPC (n = 20), oxPE (n = 21), and oxTG (n = 13) (Fig. 4i) were significantly downregulated in MB compared with normal. Together with the increase in α-ketoglutarate noted above, these lipid profiles might reflect a state of hypoxia in MB CSF because (i) cancer cells accumulate TGs due to hypoxia [62]; and (ii) hypoxia can create a deficit of glucose-derived acetyl-CoA, requiring the conversion of α-ketoglutarate into citrate so that it can be then used to generate acetyl-CoA [63]. From the practical perspective, CE, HexCer (CerG1), and SM may be promising CSF biomarkers for MB.

### Integrative analysis of transcriptome, lipidome, and metabolome

Given that the transcriptome, lipidome, and metabolome are integrated and interrelated biological systems that modulate phenotype, we next performed a multivariate analysis to integrate the molecular changes characterizing the CSF of MB patients using the data integration analysis for biomarker discovery DIABLO method in the mixOmics R package [64]. The DIABLO method identified several important features discriminating cancer from normal through interrogation of correlations between the three omics datasets.

**Fig. 5:**
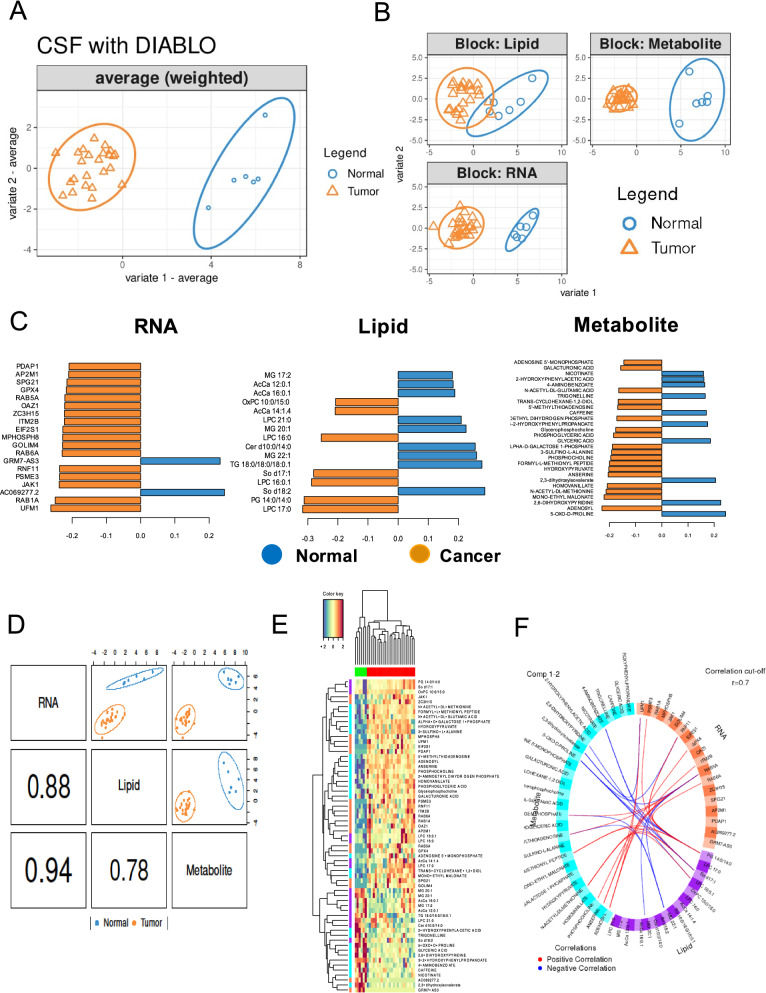
Sparse partial least-squares discriminant analysis. **A** sPLS-DA consensus plot for the combination of the three datasets showing complete discrimination of the 30 CSF samples (24 medulloblastoma and six normal samples). **B** The individual contribution of each dataset to the sPLS-DA final model, in each case showing the score plots for the two first components, indicating the best separation capability for transcriptome data followed by metabolome and lipidome data. **C** Selected features shown in pyramid bar plot. Loading plot represents the top 19 RNAs, 28 metabolites, and 16 lipids contributing to group separation. **D** Sample scatterplot from plotDiablo displaying the first component in each dataset (upper diagonal plot) and Pearson correlation between each component (lower diagonal plot). **E** Clustered image map (Euclidean distance, complete linkage) of the multi-omics signature based on the 54 multi-omics signature identified on the first component. Samples are represented in rows, selected features on the first component in columns. **F** The Circos plot (cut off: 0.7) shows positive or negative correlations denoted as red and blue lines, respectively

The first component of sparse partial least-squares discriminant analysis (sPLS-DA) [65] of the combined transcriptomic, metabolomic, and lipidomic datasets clearly discriminated normal from MB CSF samples (Fig. 5a), with the transcriptomic and metabolomic data showing the highest discriminatory capacity and correlations (Fig. 5b and Additional file 4: Fig. S6). To obtain the best discriminative features, the minimum loading coefficient for the first component of sPLS-DA was set at ± 0.15 for each data block. This filtering (Fig. 5c and d) identified n = 19 transcripts, n = 28 metabolites, and n = 16 lipids that best distinguished MB from normal samples (Fig. 5e). Among 19 RNA transcripts, ten were validated by qRT-PCR (Additional file 4: Fig. S7). The integration of data using multi-omics tools is indispensable for cancer metabolism studies [66]. Finally, to visualize the between-omics correlations in the DIABLO analysis, a Circos plot (Fig. 5f) revealed a number of strong positive and negative correlations; for example, UFM1 was positively correlated with S-adenosyl-L-methionine (Pearson's r = 0.76) and LPC 17:0 (Pearson's r = 0.6) and LPC 17:0 was positively correlated with S-adenosyl-L-methionine (Pearson's r = 0.66). UFM1 (ubiquitin-fold modifier 1) has been identified as an important factor associated with microcephaly by affecting cell cycle regulation and cancer development [67] while, in a preliminary study, S-adenosyl-L-methionine found to modulate cell cycle progression in cancer [68]. We further analyzed the MAGIC (Medulloblastoma Advanced Genomics International Consortium (https://plone.bcgsc.ca/project/magic) [7]; Additional file 4: Fig. S8 and Additional file 4: Fig. S9) datasets and found 17 out of the 19 differentially expressed RNAs in different MB subtypes (Fig. 5f).

## Conclusion

This is the first comprehensive, integrated molecular analysis of the CSF of MB patients and its comparison with normal CSF and the first to establish global transcriptomic and lipidomic profiles in the CSF of patients with MB. Our study provides proof-of-principle that all three molecular approaches can be successfully applied to CSF samples not only to discriminate MB patients from those without the disease (i.e., for biomarker discovery), but also to provide new insights into the pathobiology of the disease. Since the molecular profiles were discriminatory for the presence of MB but not the exact molecular subtype, the molecular changes in the CSF microenvironment seem to reflect general features of MB existing in that anatomical compartment. In particular, the metabolic and lipidomic profiles both contained indicators of tumor hypoxia. Our analysis provides a number of candidate biomarkers that deserve further validation, including the novel circular RNA circ_463. Due to the presence of the blood–brain barrier, CSF analysis is an ideal means to identify and assay for biomarkers arising from brain tumors that might not necessarily reach the circulation. CSF is easier to collect and less invasive than tissue biopsy and we now show that it provides a comprehensive landscape of the transcriptomic, metabolomic, and lipidomic status of MB. CSF can be used not only for primary diagnosis but also to predict responses to treatment and recurrence by monitoring biomarker levels after surgery, radiotherapy, and/or chemotherapy [69] Ideally, CSF should be collected after surgery to establish a baseline for predicting future events, and a separate CSF sample could be taken during radiographic follow-up to help establish the predictive value of these CSF biomarkers for recurrence or response to therapy [70]. Since CSF sampling is the part of standard care for patients with CNS tumors other than MB, CSF-based biomarkers hold promise for the accurate assessment of other CNS tumors.

High-throughput technologies have been used to characterize cancer in multiple dimensions including genetic, protein, transcriptomic, epigenetic, lipidomic, and metabolomic variations. Multivariate or integrative data analysis is now emerging as a powerful tool in cancer biology [71] [72]. Although it is challenging to pool independent datasets (RNA, protein, lipid, and metabolite) and combine them into one, several algorithms [72], including DIABLO used here [65], are providing robust statistical frameworks for meaningful data integration. Identifying multivariate molecular signatures in MB patients should provide information about therapeutic efficacy, disease staging, patient survival, and cancer recurrence.

Finally, it remains to be determined whether these biomarkers are sufficiently sensitive to detect recurrent disease or their optimal combination, which require further validation in prospective cohorts.

## Supplementary Information

**Additional file 1:** The genomic coordination of MB upregulated circRNAs.

**Additional file 2.:** Lipid profile of MB and normal CSF samples.

**Additional file 3:** The list of qPCR primers.

**Additional file 4.:** Supplementary tables and figures.

## Data Availability

All CSF RNA-seq datasets have been deposited in the Gene Expression Omnibus (GEO) under accession number GSE189919.

## References

[1] Ostrom QT (2020). CBTRUS statistical report: primary brain and other central nervous system tumors diagnosed in the United States in 2013–2017. Neuro Oncol.

[2] Du S (2018). Clinical characteristics and outcome of children with relapsed medulloblastoma: a retrospective study at a single center in China. J Pediatr Hematol Oncol.

[3] Srinivasan VM (2016). Modern management of medulloblastoma: Molecular classification, outcomes, and the role of surgery. Surg Neurol Int.

[4] Packer RJ (1999). Treatment of children with medulloblastomas with reduced-dose craniospinal radiation therapy and adjuvant chemotherapy: A Children's Cancer Group Study. J Clin Oncol.

[5] Weintraub L (2014). Misdiagnosing recurrent medulloblastoma: the danger of examination and imaging without histological confirmation. J Neurosurg Pediatr.

[6] Louis DN (2016). The 2016 world health organization classification of tumors of the central nervous system: a summary. Acta Neuropathol.

[7] Northcott PA (2017). The whole-genome landscape of medulloblastoma subtypes. Nature.

[8] Cavalli FMG (2017). Intertumoral Heterogeneity within Medulloblastoma Subgroups. Cancer Cell.

[9] Connolly ID (2016). The "liquid biopsy": the role of circulating DNA and RNA in central nervous system tumors. Curr Neurol Neurosci Rep.

[10] Escudero L (2020). Circulating tumour DNA from the cerebrospinal fluid allows the characterisation and monitoring of medulloblastoma. Nat Commun.

[11] Shankar GM (2017). Liquid biopsy for brain tumors. Expert Rev Mol Diagn.

[12] Wang Y (2015). Detection of tumor-derived DNA in cerebrospinal fluid of patients with primary tumors of the brain and spinal cord. Proc Natl Acad Sci U S A.

[13] Burgos KL (2013). Identification of extracellular miRNA in human cerebrospinal fluid by next-generation sequencing. RNA.

[14] Drusco A (2015). A differentially expressed set of microRNAs in cerebro-spinal fluid (CSF) can diagnose CNS malignancies. Oncotarget.

[15] Talari NK (2016). Altered tryptophan metabolism in human meningioma. J Neurooncol.

[16] Ghosh A, Nishtala K (2017). Biofluid lipidome: a source for potential diagnostic biomarkers. Clin Transl Med.

[17] Lee B (2020). Integrated RNA and metabolite profiling of urine liquid biopsies for prostate cancer biomarker discovery. Sci Rep.

[18] Folch J, Lees M, Sloane Stanley GH (1957). A simple method for the isolation and purification of total lipides from animal tissues. J Biol Chem.

[19] Chen S (2018). fastp: an ultra-fast all-in-one FASTQ preprocessor. Bioinformatics.

[20] Dobin A (2013). STAR: ultrafast universal RNA-seq aligner. Bioinformatics (Oxford, England).

[21] Liao Y, Smyth GK, Shi W (2014). featureCounts: an efficient general purpose program for assigning sequence reads to genomic features. Bioinformatics.

[22] Law CW, et al (2016) RNA-seq analysis is easy as 1–2–3 with limma, Glimma and edgeR*.* F1000Res, 5 10.12688/f1000research.9005.1 PMC4937821 27441086

[23] Ritchie ME (2015). limma powers differential expression analyses for RNA-sequencing and microarray studies. Nucleic Acids Res.

[24] Langmead B, Salzberg SL (2012). Fast gapped-read alignment with Bowtie 2. Nat Methods.

[25] Gao Y, Zhang J, Zhao F (2018). Circular RNA identification based on multiple seed matching. Brief Bioinform.

[26] Hansen TB (2018). Improved circRNA Identification by combining prediction algorithms. Front Cell Dev Biol.

[27] Love MI, Huber W, Anders S (2014). Moderated estimation of fold change and dispersion for RNA-seq data with DESeq2. Genome Biol.

[28] Pluskal T (2010). MZmine 2: modular framework for processing, visualizing, and analyzing mass spectrometry-based molecular profile data. BMC Bioinform.

[29] Koelmel JP (2017). LipidMatch: an automated workflow for rule-based lipid identification using untargeted high-resolution tandem mass spectrometry data. BMC Bioinform.

[30] Pang, Z., et al., *MetaboAnalyst 5.0: narrowing the gap between raw spectra and functional insights.* Nucleic Acids Res, 2021. **49**(W1): p. W388-W396. 10.1093/nar/gkab382 PMC8265181 34019663

[31] Rohart F (2017). mixOmics: An R package for 'omics feature selection and multiple data integration. PLoS Comput Biol.

[32] Li J, et al (2020) Reliable tumor detection by whole-genome methylation sequencing of cell-free DNA in cerebrospinal fluid of pediatric medulloblastoma*.* Sci Adv **6**(42) 10.1126/sciadv.abb5427 PMC7567591 33067228

[33] De Mattos-Arruda L (2015). Cerebrospinal fluid-derived circulating tumour DNA better represents the genomic alterations of brain tumours than plasma. Nat Commun.

[34] Hulstaert E (2020). Charting extracellular transcriptomes in the human biofluid RNA atlas. Cell Rep.

[35] Subramanian A (2005). Gene set enrichment analysis: a knowledge-based approach for interpreting genome-wide expression profiles. Proc Natl Acad Sci U S A.

[36] Aref D (2013). Canonical TGF-beta pathway activity is a predictor of SHH-driven medulloblastoma survival and delineates putative precursors in cerebellar development. Brain Pathol.

[37] Gate D (2014). T-cell TGF-beta signaling abrogation restricts medulloblastoma progression. Proc Natl Acad Sci U S A.

[38] Li HM, Ma XL, Li HG (2019). Intriguing circles: conflicts and controversies in circular RNA research. Wiley Interdiscip Rev RNA.

[39] Su M (2019). Circular RNAs in Cancer: emerging functions in hallmarks, stemness, resistance and roles as potential biomarkers. Mol Cancer.

[40] Li S (2018). exoRBase: a database of circRNA, lncRNA and mRNA in human blood exosomes. Nucleic Acids Res.

[41] Bahn JH (2015). The landscape of microRNA, Piwi-interacting RNA, and circular RNA in human saliva. Clin Chem.

[42] Liu B (2019). Characterization of tissue-specific biomarkers with the expression of circRNAs in forensically relevant body fluids. Int J Legal Med.

[43] Kolling M (2019). Circular RNAs in urine of kidney transplant patients with acute T cell-mediated allograft rejection. Clin Chem.

[44] Hansen TB (2011). miRNA-dependent gene silencing involving Ago2-mediated cleavage of a circular antisense RNA. EMBO J.

[45] Hansen TB (2013). Natural RNA circles function as efficient microRNA sponges. Nature.

[46] Li RC (2018). CiRS-7 promotes growth and metastasis of esophageal squamous cell carcinoma via regulation of miR-7/HOXB13. Cell Death Dis.

[47] Hanniford D (2020). Epigenetic silencing of CDR1as drives IGF2BP3-mediated melanoma invasion and metastasis. Cancer Cell.

[48] Jakobsen T, et al. (2021) Genome-wide circular RNA expression patterns reflect resistance to immunomodulatory drugs in multiple myeloma cells*.* Cancers (Basel), 13(3) 10.3390/cancers13030365 PMC7930955 33498476

[49] Lv T (2018). Dysregulated circular RNAs in medulloblastoma regulate proliferation and growth of tumor cells via host genes. Cancer Med.

[50] Zhao X, Guan J, Luo M (2021). Circ-SKA3 upregulates ID3 expression by decoying miR-326 to accelerate the development of medulloblastoma. J Clin Neurosci.

[51] Rickert D (2021). Circular RNA profiling distinguishes medulloblastoma groups and shows aberrant RMST overexpression in WNT medulloblastoma. Acta Neuropathol.

[52] Tan SK (2021). Obesity-dependent adipokine chemerin suppresses fatty acid oxidation to confer ferroptosis resistance. Cancer Discov.

[53] Pavlova NN, Thompson CB (2016). The emerging hallmarks of cancer metabolism. Cell Metab.

[54] Tech K, Deshmukh M, Gershon TR (2015) Adaptations of energy metabolism during cerebellar neurogenesis are co-opted in medulloblastoma. Cancer Lett 356(2 Pt A): 268–72 10.1016/j.canlet.2014.02.017 PMC4141892 24569090

[55] Ballester LY (2018). Analysis of cerebrospinal fluid metabolites in patients with primary or metastatic central nervous system tumors. Acta Neuropathol Commun.

[56] Reichl B et al. (2020) Determination of a tumor-promoting microenvironment in recurrent medulloblastoma: a multi-omics study of cerebrospinal fluid*.* Cancers (Basel), 12(6) 10.3390/cancers12061350 PMC7352284 32466393

[57] DeBerardinis RJ (2007). Beyond aerobic glycolysis: transformed cells can engage in glutamine metabolism that exceeds the requirement for protein and nucleotide synthesis. Proc Natl Acad Sci U S A.

[58] Son J (2013). Glutamine supports pancreatic cancer growth through a KRAS-regulated metabolic pathway. Nature.

[59] Mahmud I, Garrett TJ (2020). Lipidomics in human cancer and malnutrition. New techniques for studying biomembranes.

[60] Bennett CD (2018). Tissue metabolite profiles for the characterisation of paediatric cerebellar tumours. Sci Rep.

[61] Huang D (2020). Lipidome signatures of metastasis in a transgenic mouse model of sonic hedgehog medulloblastoma. Anal Bioanal Chem.

[62] Mylonis I (2012). Hypoxia causes triglyceride accumulation by HIF-1-mediated stimulation of lipin 1 expression. J Cell Sci.

[63] Wise DR (2011). Hypoxia promotes isocitrate dehydrogenase-dependent carboxylation of alpha-ketoglutarate to citrate to support cell growth and viability. Proc Natl Acad Sci U S A.

[64] Rohart F (2017). mixOmics: An R package for 'omics feature selection and multiple data integration. PLoS Comput Biol.

[65] Le Cao KA, Boitard S, Besse P (2011). Sparse PLS discriminant analysis: biologically relevant feature selection and graphical displays for multiclass problems. BMC Bioinform.

[66] Cantor JR, Sabatini DM (2012). Cancer cell metabolism: one hallmark, many faces. Cancer Discov.

[67] Yu L (2020). The UFM1 cascade times mitosis entry associated with microcephaly. FASEB J.

[68] Mosca L (2020). Effects of SadenosylLmethionine on the invasion and migration of head and neck squamous cancer cells and analysis of the underlying mechanisms. Int J Oncol.

[69] Zorofchian S (2019). Circulating tumour DNA, microRNA and metabolites in cerebrospinal fluid as biomarkers for central nervous system malignancies. J Clin Pathol.

[70] Mattox AK, Yan H, Bettegowda C (2019). The potential of cerebrospinal fluid-based liquid biopsy approaches in CNS tumors. Neuro Oncol.

[71] Liang M (2015). Integrative data analysis of multi-platform cancer data with a multimodal deep learning approach. IEEE/ACM Trans Comput Biol Bioinform.

[72] Pavel AB, Sonkin D, Reddy A (2016). Integrative modeling of multi-omics data to identify cancer drivers and infer patient-specific gene activity. BMC Syst Biol.

