# Correction to: Medulloblastoma cerebrospinal fluid reveals metabolites and lipids indicative of hypoxia and cancer-specific RNAs

**DOI:** 10.1186/s40478-022-01368-x

**Published:** 2022-04-22

**Authors:** Bongyong Lee, Iqbal Mahmud, Rudramani Pokhrel, Rabi Murad, Menglang Yuan, Stacie Stapleton, Chetan Bettegowda, George Jallo, Charles G. Eberhart, Timothy Garrett, Ranjan J. Perera

**Affiliations:** 1 Department of Oncology, Sidney Kimmel Comprehensive Cancer Center, School of Medicine, Johns Hopkins University, 1650 Orleans St, Baltimore, MD 21231 USA; 2 Johns Hopkins All Children’s Hospital, 600 5th St. South, St. Petersburg, FL 33701 USA; 3 Department Pathology, Immunology and Laboratory Medicine, College of Medicine, University of Florida, 1395 Center Drive, Gainesville, FL 32610 USA; 4 Sanford Burnham Prebys Medical Discovery Institute, 10901 N. Torrey Pines Road, La Jolla, CA 92037 USA; 5 Department of Neurosurgery, Johns Hopkins University School of Medicine, Baltimore, USA; 6 Department of Pathology, Johns Hopkins University School of Medicine, 720 Rutland Avenue, Baltimore, MD 21205 USA; 7 Department of Bioinformatics and Computational Biology, The University of Texas MD Anderson Cancer Center, Houston, TX 77030 USA

## Correction to: Acta Neuropathologica Communications (2022) 10:25 https://doi.org/10.1186/s40478-022-01326-7

Following publication of the original article [1], the authors identified an error in the author name of Iqbal Mahmud.

The incorrect author name is: Iqbal Mohamad

The correct author name is: Iqbal Mahmud

In addition, the authors identified an error in Fig. 1, panel B. The correct figure is given below.

**Fig. 1:**
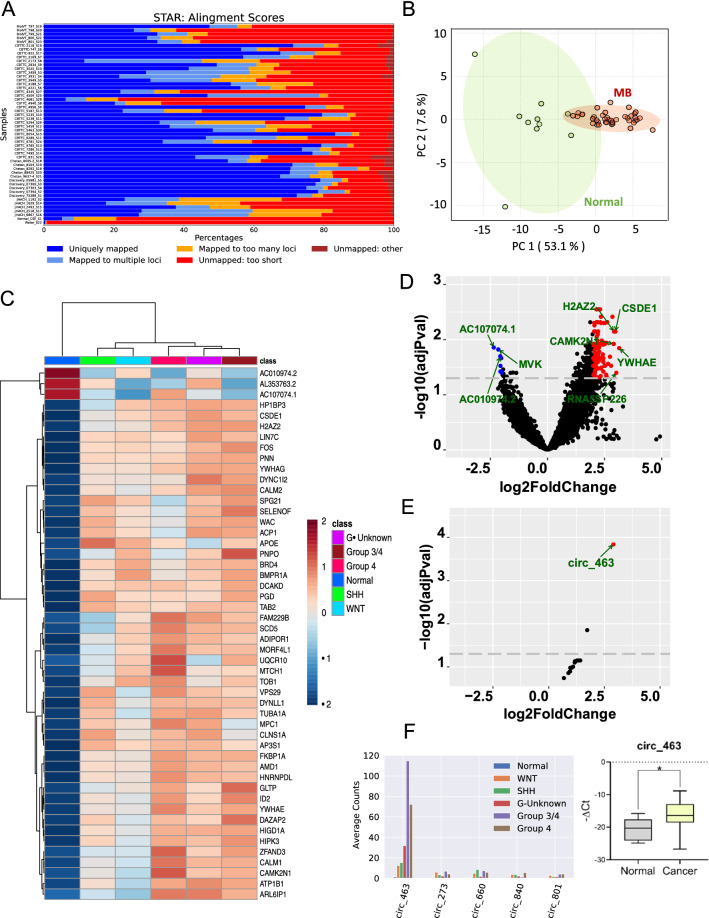
Global transcriptomic differences in the CSF of patients with (n = 40) and without (n = 11) MB. **A** Mapping rate of each CSF sample. **B** Principal component analysis of CSF samples using the 48 most differentially expressed genes showing clear separation of normal CSF samples from MB CSF samples. **C** Unsupervised clustering of samples using the 48 most differentially expressed genes showing clear separation of normal CSF samples from MB CSF samples. **D** Volcano plot showing significantly up- or downregulated genes in CSF. **E** Volcano plot for differentially expressed circRNAs between normal vs MB CSF samples. **F** Top 5 circRNAs expression in different subgroups and qRT-PCR validation of circ-463

The original article [1] has been corrected.

## References

[1] Lee B (2022). Medulloblastoma cerebrospinal fluid reveals metabolites and lipids indicative of hypoxia and cancer-specific RNAs. Acta Neuropathol Commun.

