# Supplementary material for: Medulloblastoma cerebrospinal fluid reveals metabolites and lipids indicative of hypoxia and cancer-specific RNAs
Source: Acta Neuropathol Commun. 2022 Feb 24;10:25. doi: 10.1186/s40478-022-01326-7 (PMC8867780; doi:10.1186/s40478-022-01326-7)

## Slide 1
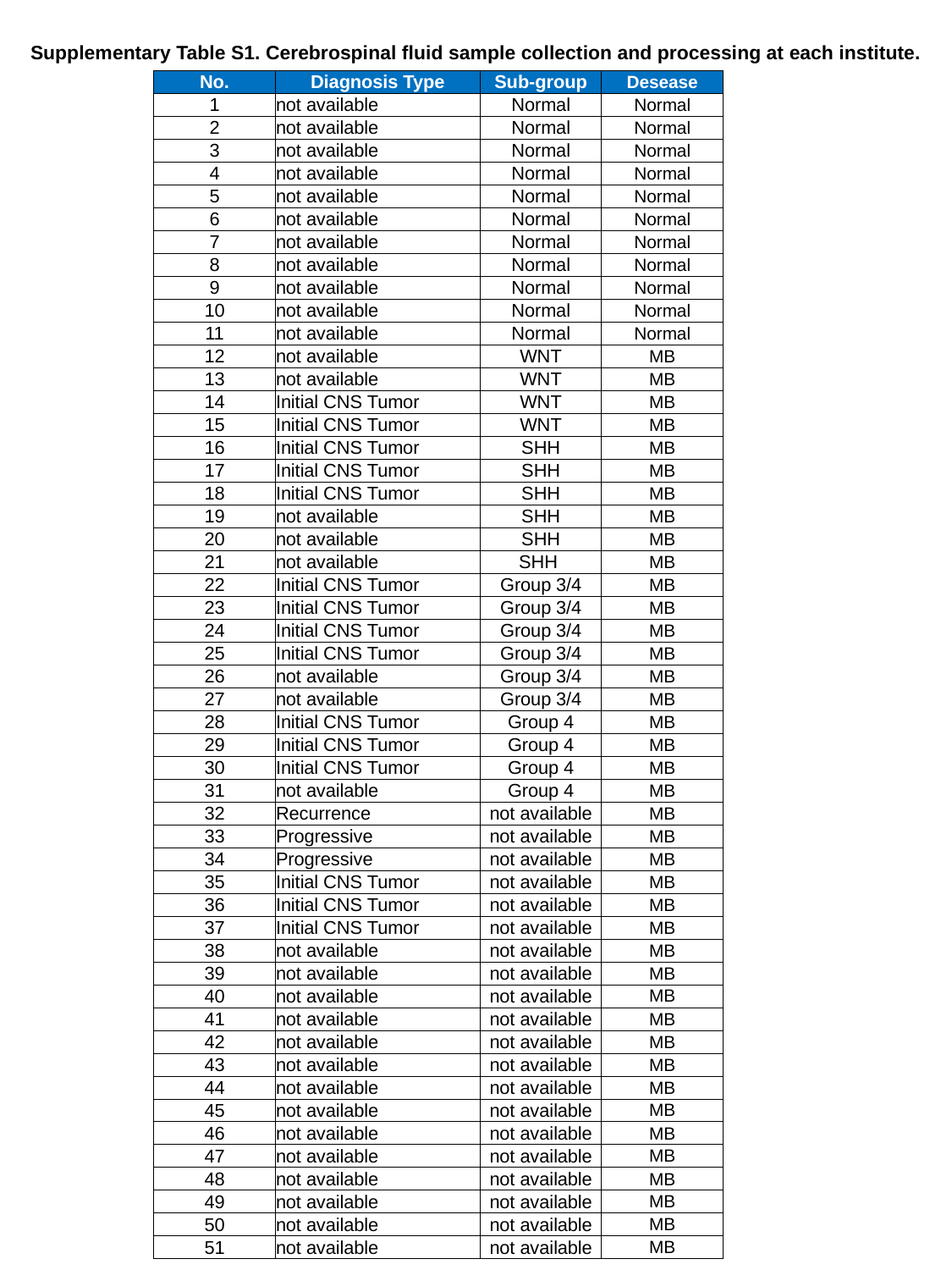

Supplementary Table S1. Cerebrospinal fluid sample collection and processing at each institute.
| No. | Diagnosis Type | Sub-group | Desease |
| --- | --- | --- | --- |
| 1 | not available | Normal | Normal |
| 2 | not available | Normal | Normal |
| 3 | not available | Normal | Normal |
| 4 | not available | Normal | Normal |
| 5 | not available | Normal | Normal |
| 6 | not available | Normal | Normal |
| 7 | not available | Normal | Normal |
| 8 | not available | Normal | Normal |
| 9 | not available | Normal | Normal |
| 10 | not available | Normal | Normal |
| 11 | not available | Normal | Normal |
| 12 | not available | WNT | MB |
| 13 | not available | WNT | MB |
| 14 | Initial CNS Tumor | WNT | MB |
| 15 | Initial CNS Tumor | WNT | MB |
| 16 | Initial CNS Tumor | SHH | MB |
| 17 | Initial CNS Tumor | SHH | MB |
| 18 | Initial CNS Tumor | SHH | MB |
| 19 | not available | SHH | MB |
| 20 | not available | SHH | MB |
| 21 | not available | SHH | MB |
| 22 | Initial CNS Tumor | Group 3/4 | MB |
| 23 | Initial CNS Tumor | Group 3/4 | MB |
| 24 | Initial CNS Tumor | Group 3/4 | MB |
| 25 | Initial CNS Tumor | Group 3/4 | MB |
| 26 | not available | Group 3/4 | MB |
| 27 | not available | Group 3/4 | MB |
| 28 | Initial CNS Tumor | Group 4 | MB |
| 29 | Initial CNS Tumor | Group 4 | MB |
| 30 | Initial CNS Tumor | Group 4 | MB |
| 31 | not available | Group 4 | MB |
| 32 | Recurrence | not available | MB |
| 33 | Progressive | not available | MB |
| 34 | Progressive | not available | MB |
| 35 | Initial CNS Tumor | not available | MB |
| 36 | Initial CNS Tumor | not available | MB |
| 37 | Initial CNS Tumor | not available | MB |
| 38 | not available | not available | MB |
| 39 | not available | not available | MB |
| 40 | not available | not available | MB |
| 41 | not available | not available | MB |
| 42 | not available | not available | MB |
| 43 | not available | not available | MB |
| 44 | not available | not available | MB |
| 45 | not available | not available | MB |
| 46 | not available | not available | MB |
| 47 | not available | not available | MB |
| 48 | not available | not available | MB |
| 49 | not available | not available | MB |
| 50 | not available | not available | MB |
| 51 | not available | not available | MB |

## Slide 2
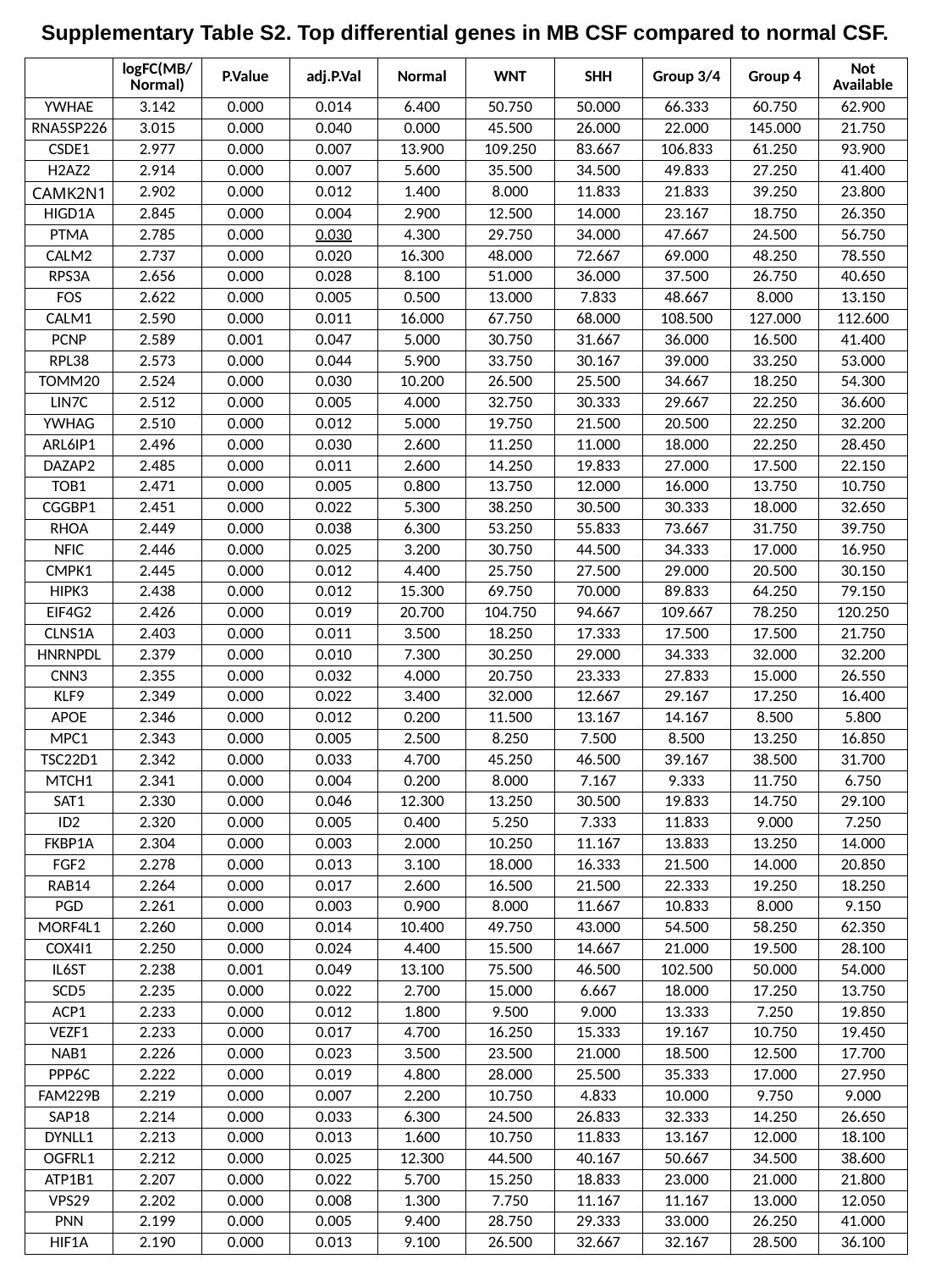

Supplementary Table S2. Top differential genes in MB CSF compared to normal CSF.
| | logFC(MB/Normal) | P.Value | adj.P.Val | Normal | WNT | SHH | Group 3/4 | Group 4 | Not Available |
| --- | --- | --- | --- | --- | --- | --- | --- | --- | --- |
| YWHAE | 3.142 | 0.000 | 0.014 | 6.400 | 50.750 | 50.000 | 66.333 | 60.750 | 62.900 |
| RNA5SP226 | 3.015 | 0.000 | 0.040 | 0.000 | 45.500 | 26.000 | 22.000 | 145.000 | 21.750 |
| CSDE1 | 2.977 | 0.000 | 0.007 | 13.900 | 109.250 | 83.667 | 106.833 | 61.250 | 93.900 |
| H2AZ2 | 2.914 | 0.000 | 0.007 | 5.600 | 35.500 | 34.500 | 49.833 | 27.250 | 41.400 |
| CAMK2N1 | 2.902 | 0.000 | 0.012 | 1.400 | 8.000 | 11.833 | 21.833 | 39.250 | 23.800 |
| HIGD1A | 2.845 | 0.000 | 0.004 | 2.900 | 12.500 | 14.000 | 23.167 | 18.750 | 26.350 |
| PTMA | 2.785 | 0.000 | 0.030 | 4.300 | 29.750 | 34.000 | 47.667 | 24.500 | 56.750 |
| CALM2 | 2.737 | 0.000 | 0.020 | 16.300 | 48.000 | 72.667 | 69.000 | 48.250 | 78.550 |
| RPS3A | 2.656 | 0.000 | 0.028 | 8.100 | 51.000 | 36.000 | 37.500 | 26.750 | 40.650 |
| FOS | 2.622 | 0.000 | 0.005 | 0.500 | 13.000 | 7.833 | 48.667 | 8.000 | 13.150 |
| CALM1 | 2.590 | 0.000 | 0.011 | 16.000 | 67.750 | 68.000 | 108.500 | 127.000 | 112.600 |
| PCNP | 2.589 | 0.001 | 0.047 | 5.000 | 30.750 | 31.667 | 36.000 | 16.500 | 41.400 |
| RPL38 | 2.573 | 0.000 | 0.044 | 5.900 | 33.750 | 30.167 | 39.000 | 33.250 | 53.000 |
| TOMM20 | 2.524 | 0.000 | 0.030 | 10.200 | 26.500 | 25.500 | 34.667 | 18.250 | 54.300 |
| LIN7C | 2.512 | 0.000 | 0.005 | 4.000 | 32.750 | 30.333 | 29.667 | 22.250 | 36.600 |
| YWHAG | 2.510 | 0.000 | 0.012 | 5.000 | 19.750 | 21.500 | 20.500 | 22.250 | 32.200 |
| ARL6IP1 | 2.496 | 0.000 | 0.030 | 2.600 | 11.250 | 11.000 | 18.000 | 22.250 | 28.450 |
| DAZAP2 | 2.485 | 0.000 | 0.011 | 2.600 | 14.250 | 19.833 | 27.000 | 17.500 | 22.150 |
| TOB1 | 2.471 | 0.000 | 0.005 | 0.800 | 13.750 | 12.000 | 16.000 | 13.750 | 10.750 |
| CGGBP1 | 2.451 | 0.000 | 0.022 | 5.300 | 38.250 | 30.500 | 30.333 | 18.000 | 32.650 |
| RHOA | 2.449 | 0.000 | 0.038 | 6.300 | 53.250 | 55.833 | 73.667 | 31.750 | 39.750 |
| NFIC | 2.446 | 0.000 | 0.025 | 3.200 | 30.750 | 44.500 | 34.333 | 17.000 | 16.950 |
| CMPK1 | 2.445 | 0.000 | 0.012 | 4.400 | 25.750 | 27.500 | 29.000 | 20.500 | 30.150 |
| HIPK3 | 2.438 | 0.000 | 0.012 | 15.300 | 69.750 | 70.000 | 89.833 | 64.250 | 79.150 |
| EIF4G2 | 2.426 | 0.000 | 0.019 | 20.700 | 104.750 | 94.667 | 109.667 | 78.250 | 120.250 |
| CLNS1A | 2.403 | 0.000 | 0.011 | 3.500 | 18.250 | 17.333 | 17.500 | 17.500 | 21.750 |
| HNRNPDL | 2.379 | 0.000 | 0.010 | 7.300 | 30.250 | 29.000 | 34.333 | 32.000 | 32.200 |
| CNN3 | 2.355 | 0.000 | 0.032 | 4.000 | 20.750 | 23.333 | 27.833 | 15.000 | 26.550 |
| KLF9 | 2.349 | 0.000 | 0.022 | 3.400 | 32.000 | 12.667 | 29.167 | 17.250 | 16.400 |
| APOE | 2.346 | 0.000 | 0.012 | 0.200 | 11.500 | 13.167 | 14.167 | 8.500 | 5.800 |
| MPC1 | 2.343 | 0.000 | 0.005 | 2.500 | 8.250 | 7.500 | 8.500 | 13.250 | 16.850 |
| TSC22D1 | 2.342 | 0.000 | 0.033 | 4.700 | 45.250 | 46.500 | 39.167 | 38.500 | 31.700 |
| MTCH1 | 2.341 | 0.000 | 0.004 | 0.200 | 8.000 | 7.167 | 9.333 | 11.750 | 6.750 |
| SAT1 | 2.330 | 0.000 | 0.046 | 12.300 | 13.250 | 30.500 | 19.833 | 14.750 | 29.100 |
| ID2 | 2.320 | 0.000 | 0.005 | 0.400 | 5.250 | 7.333 | 11.833 | 9.000 | 7.250 |
| FKBP1A | 2.304 | 0.000 | 0.003 | 2.000 | 10.250 | 11.167 | 13.833 | 13.250 | 14.000 |
| FGF2 | 2.278 | 0.000 | 0.013 | 3.100 | 18.000 | 16.333 | 21.500 | 14.000 | 20.850 |
| RAB14 | 2.264 | 0.000 | 0.017 | 2.600 | 16.500 | 21.500 | 22.333 | 19.250 | 18.250 |
| PGD | 2.261 | 0.000 | 0.003 | 0.900 | 8.000 | 11.667 | 10.833 | 8.000 | 9.150 |
| MORF4L1 | 2.260 | 0.000 | 0.014 | 10.400 | 49.750 | 43.000 | 54.500 | 58.250 | 62.350 |
| COX4I1 | 2.250 | 0.000 | 0.024 | 4.400 | 15.500 | 14.667 | 21.000 | 19.500 | 28.100 |
| IL6ST | 2.238 | 0.001 | 0.049 | 13.100 | 75.500 | 46.500 | 102.500 | 50.000 | 54.000 |
| SCD5 | 2.235 | 0.000 | 0.022 | 2.700 | 15.000 | 6.667 | 18.000 | 17.250 | 13.750 |
| ACP1 | 2.233 | 0.000 | 0.012 | 1.800 | 9.500 | 9.000 | 13.333 | 7.250 | 19.850 |
| VEZF1 | 2.233 | 0.000 | 0.017 | 4.700 | 16.250 | 15.333 | 19.167 | 10.750 | 19.450 |
| NAB1 | 2.226 | 0.000 | 0.023 | 3.500 | 23.500 | 21.000 | 18.500 | 12.500 | 17.700 |
| PPP6C | 2.222 | 0.000 | 0.019 | 4.800 | 28.000 | 25.500 | 35.333 | 17.000 | 27.950 |
| FAM229B | 2.219 | 0.000 | 0.007 | 2.200 | 10.750 | 4.833 | 10.000 | 9.750 | 9.000 |
| SAP18 | 2.214 | 0.000 | 0.033 | 6.300 | 24.500 | 26.833 | 32.333 | 14.250 | 26.650 |
| DYNLL1 | 2.213 | 0.000 | 0.013 | 1.600 | 10.750 | 11.833 | 13.167 | 12.000 | 18.100 |
| OGFRL1 | 2.212 | 0.000 | 0.025 | 12.300 | 44.500 | 40.167 | 50.667 | 34.500 | 38.600 |
| ATP1B1 | 2.207 | 0.000 | 0.022 | 5.700 | 15.250 | 18.833 | 23.000 | 21.000 | 21.800 |
| VPS29 | 2.202 | 0.000 | 0.008 | 1.300 | 7.750 | 11.167 | 11.167 | 13.000 | 12.050 |
| PNN | 2.199 | 0.000 | 0.005 | 9.400 | 28.750 | 29.333 | 33.000 | 26.250 | 41.000 |
| HIF1A | 2.190 | 0.000 | 0.013 | 9.100 | 26.500 | 32.667 | 32.167 | 28.500 | 36.100 |

## Slide 3
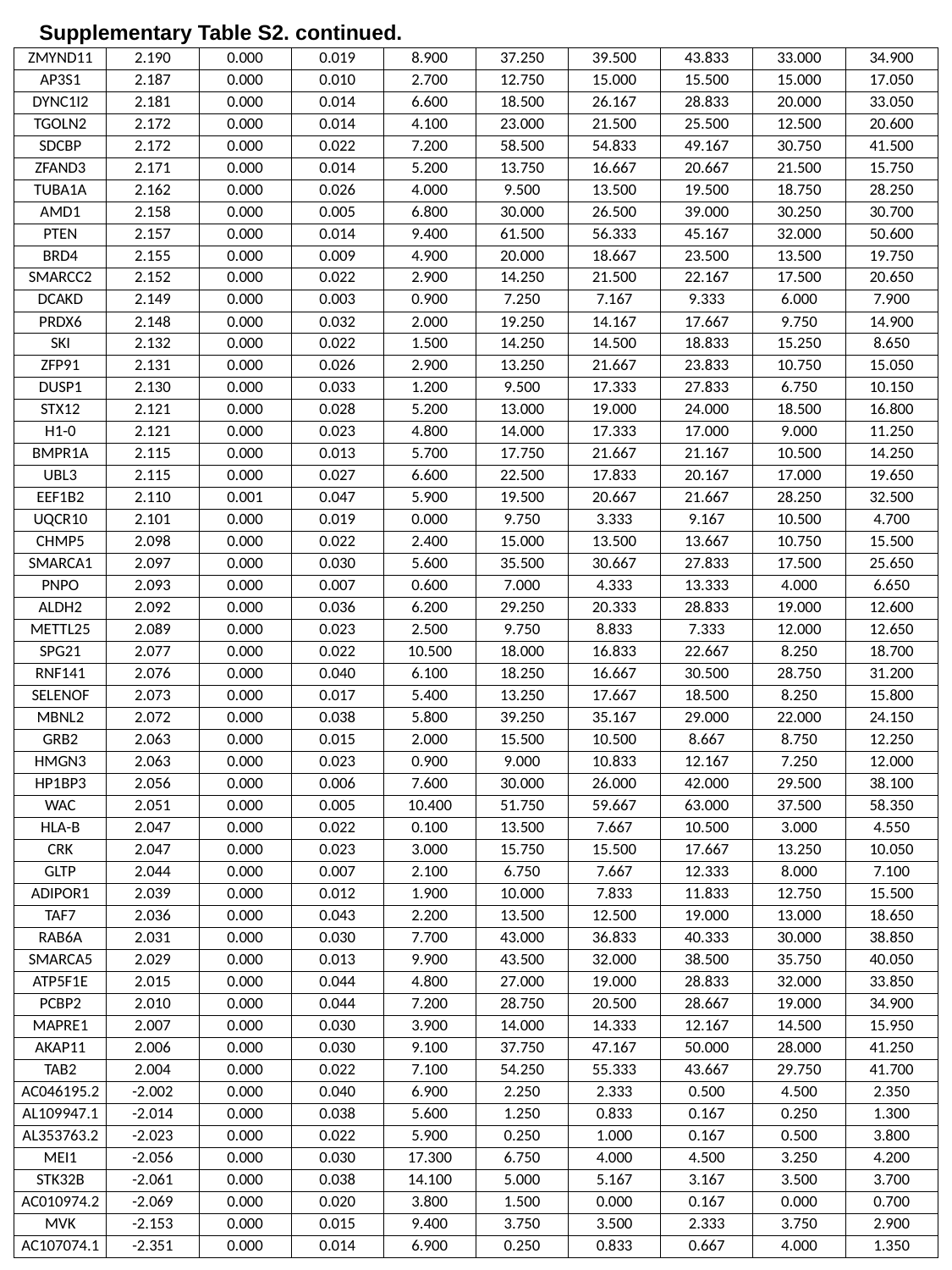

Supplementary Table S2. continued.
| ZMYND11 | 2.190 | 0.000 | 0.019 | 8.900 | 37.250 | 39.500 | 43.833 | 33.000 | 34.900 |
| --- | --- | --- | --- | --- | --- | --- | --- | --- | --- |
| AP3S1 | 2.187 | 0.000 | 0.010 | 2.700 | 12.750 | 15.000 | 15.500 | 15.000 | 17.050 |
| DYNC1I2 | 2.181 | 0.000 | 0.014 | 6.600 | 18.500 | 26.167 | 28.833 | 20.000 | 33.050 |
| TGOLN2 | 2.172 | 0.000 | 0.014 | 4.100 | 23.000 | 21.500 | 25.500 | 12.500 | 20.600 |
| SDCBP | 2.172 | 0.000 | 0.022 | 7.200 | 58.500 | 54.833 | 49.167 | 30.750 | 41.500 |
| ZFAND3 | 2.171 | 0.000 | 0.014 | 5.200 | 13.750 | 16.667 | 20.667 | 21.500 | 15.750 |
| TUBA1A | 2.162 | 0.000 | 0.026 | 4.000 | 9.500 | 13.500 | 19.500 | 18.750 | 28.250 |
| AMD1 | 2.158 | 0.000 | 0.005 | 6.800 | 30.000 | 26.500 | 39.000 | 30.250 | 30.700 |
| PTEN | 2.157 | 0.000 | 0.014 | 9.400 | 61.500 | 56.333 | 45.167 | 32.000 | 50.600 |
| BRD4 | 2.155 | 0.000 | 0.009 | 4.900 | 20.000 | 18.667 | 23.500 | 13.500 | 19.750 |
| SMARCC2 | 2.152 | 0.000 | 0.022 | 2.900 | 14.250 | 21.500 | 22.167 | 17.500 | 20.650 |
| DCAKD | 2.149 | 0.000 | 0.003 | 0.900 | 7.250 | 7.167 | 9.333 | 6.000 | 7.900 |
| PRDX6 | 2.148 | 0.000 | 0.032 | 2.000 | 19.250 | 14.167 | 17.667 | 9.750 | 14.900 |
| SKI | 2.132 | 0.000 | 0.022 | 1.500 | 14.250 | 14.500 | 18.833 | 15.250 | 8.650 |
| ZFP91 | 2.131 | 0.000 | 0.026 | 2.900 | 13.250 | 21.667 | 23.833 | 10.750 | 15.050 |
| DUSP1 | 2.130 | 0.000 | 0.033 | 1.200 | 9.500 | 17.333 | 27.833 | 6.750 | 10.150 |
| STX12 | 2.121 | 0.000 | 0.028 | 5.200 | 13.000 | 19.000 | 24.000 | 18.500 | 16.800 |
| H1-0 | 2.121 | 0.000 | 0.023 | 4.800 | 14.000 | 17.333 | 17.000 | 9.000 | 11.250 |
| BMPR1A | 2.115 | 0.000 | 0.013 | 5.700 | 17.750 | 21.667 | 21.167 | 10.500 | 14.250 |
| UBL3 | 2.115 | 0.000 | 0.027 | 6.600 | 22.500 | 17.833 | 20.167 | 17.000 | 19.650 |
| EEF1B2 | 2.110 | 0.001 | 0.047 | 5.900 | 19.500 | 20.667 | 21.667 | 28.250 | 32.500 |
| UQCR10 | 2.101 | 0.000 | 0.019 | 0.000 | 9.750 | 3.333 | 9.167 | 10.500 | 4.700 |
| CHMP5 | 2.098 | 0.000 | 0.022 | 2.400 | 15.000 | 13.500 | 13.667 | 10.750 | 15.500 |
| SMARCA1 | 2.097 | 0.000 | 0.030 | 5.600 | 35.500 | 30.667 | 27.833 | 17.500 | 25.650 |
| PNPO | 2.093 | 0.000 | 0.007 | 0.600 | 7.000 | 4.333 | 13.333 | 4.000 | 6.650 |
| ALDH2 | 2.092 | 0.000 | 0.036 | 6.200 | 29.250 | 20.333 | 28.833 | 19.000 | 12.600 |
| METTL25 | 2.089 | 0.000 | 0.023 | 2.500 | 9.750 | 8.833 | 7.333 | 12.000 | 12.650 |
| SPG21 | 2.077 | 0.000 | 0.022 | 10.500 | 18.000 | 16.833 | 22.667 | 8.250 | 18.700 |
| RNF141 | 2.076 | 0.000 | 0.040 | 6.100 | 18.250 | 16.667 | 30.500 | 28.750 | 31.200 |
| SELENOF | 2.073 | 0.000 | 0.017 | 5.400 | 13.250 | 17.667 | 18.500 | 8.250 | 15.800 |
| MBNL2 | 2.072 | 0.000 | 0.038 | 5.800 | 39.250 | 35.167 | 29.000 | 22.000 | 24.150 |
| GRB2 | 2.063 | 0.000 | 0.015 | 2.000 | 15.500 | 10.500 | 8.667 | 8.750 | 12.250 |
| HMGN3 | 2.063 | 0.000 | 0.023 | 0.900 | 9.000 | 10.833 | 12.167 | 7.250 | 12.000 |
| HP1BP3 | 2.056 | 0.000 | 0.006 | 7.600 | 30.000 | 26.000 | 42.000 | 29.500 | 38.100 |
| WAC | 2.051 | 0.000 | 0.005 | 10.400 | 51.750 | 59.667 | 63.000 | 37.500 | 58.350 |
| HLA-B | 2.047 | 0.000 | 0.022 | 0.100 | 13.500 | 7.667 | 10.500 | 3.000 | 4.550 |
| CRK | 2.047 | 0.000 | 0.023 | 3.000 | 15.750 | 15.500 | 17.667 | 13.250 | 10.050 |
| GLTP | 2.044 | 0.000 | 0.007 | 2.100 | 6.750 | 7.667 | 12.333 | 8.000 | 7.100 |
| ADIPOR1 | 2.039 | 0.000 | 0.012 | 1.900 | 10.000 | 7.833 | 11.833 | 12.750 | 15.500 |
| TAF7 | 2.036 | 0.000 | 0.043 | 2.200 | 13.500 | 12.500 | 19.000 | 13.000 | 18.650 |
| RAB6A | 2.031 | 0.000 | 0.030 | 7.700 | 43.000 | 36.833 | 40.333 | 30.000 | 38.850 |
| SMARCA5 | 2.029 | 0.000 | 0.013 | 9.900 | 43.500 | 32.000 | 38.500 | 35.750 | 40.050 |
| ATP5F1E | 2.015 | 0.000 | 0.044 | 4.800 | 27.000 | 19.000 | 28.833 | 32.000 | 33.850 |
| PCBP2 | 2.010 | 0.000 | 0.044 | 7.200 | 28.750 | 20.500 | 28.667 | 19.000 | 34.900 |
| MAPRE1 | 2.007 | 0.000 | 0.030 | 3.900 | 14.000 | 14.333 | 12.167 | 14.500 | 15.950 |
| AKAP11 | 2.006 | 0.000 | 0.030 | 9.100 | 37.750 | 47.167 | 50.000 | 28.000 | 41.250 |
| TAB2 | 2.004 | 0.000 | 0.022 | 7.100 | 54.250 | 55.333 | 43.667 | 29.750 | 41.700 |
| AC046195.2 | -2.002 | 0.000 | 0.040 | 6.900 | 2.250 | 2.333 | 0.500 | 4.500 | 2.350 |
| AL109947.1 | -2.014 | 0.000 | 0.038 | 5.600 | 1.250 | 0.833 | 0.167 | 0.250 | 1.300 |
| AL353763.2 | -2.023 | 0.000 | 0.022 | 5.900 | 0.250 | 1.000 | 0.167 | 0.500 | 3.800 |
| MEI1 | -2.056 | 0.000 | 0.030 | 17.300 | 6.750 | 4.000 | 4.500 | 3.250 | 4.200 |
| STK32B | -2.061 | 0.000 | 0.038 | 14.100 | 5.000 | 5.167 | 3.167 | 3.500 | 3.700 |
| AC010974.2 | -2.069 | 0.000 | 0.020 | 3.800 | 1.500 | 0.000 | 0.167 | 0.000 | 0.700 |
| MVK | -2.153 | 0.000 | 0.015 | 9.400 | 3.750 | 3.500 | 2.333 | 3.750 | 2.900 |
| AC107074.1 | -2.351 | 0.000 | 0.014 | 6.900 | 0.250 | 0.833 | 0.667 | 4.000 | 1.350 |

## Slide 4
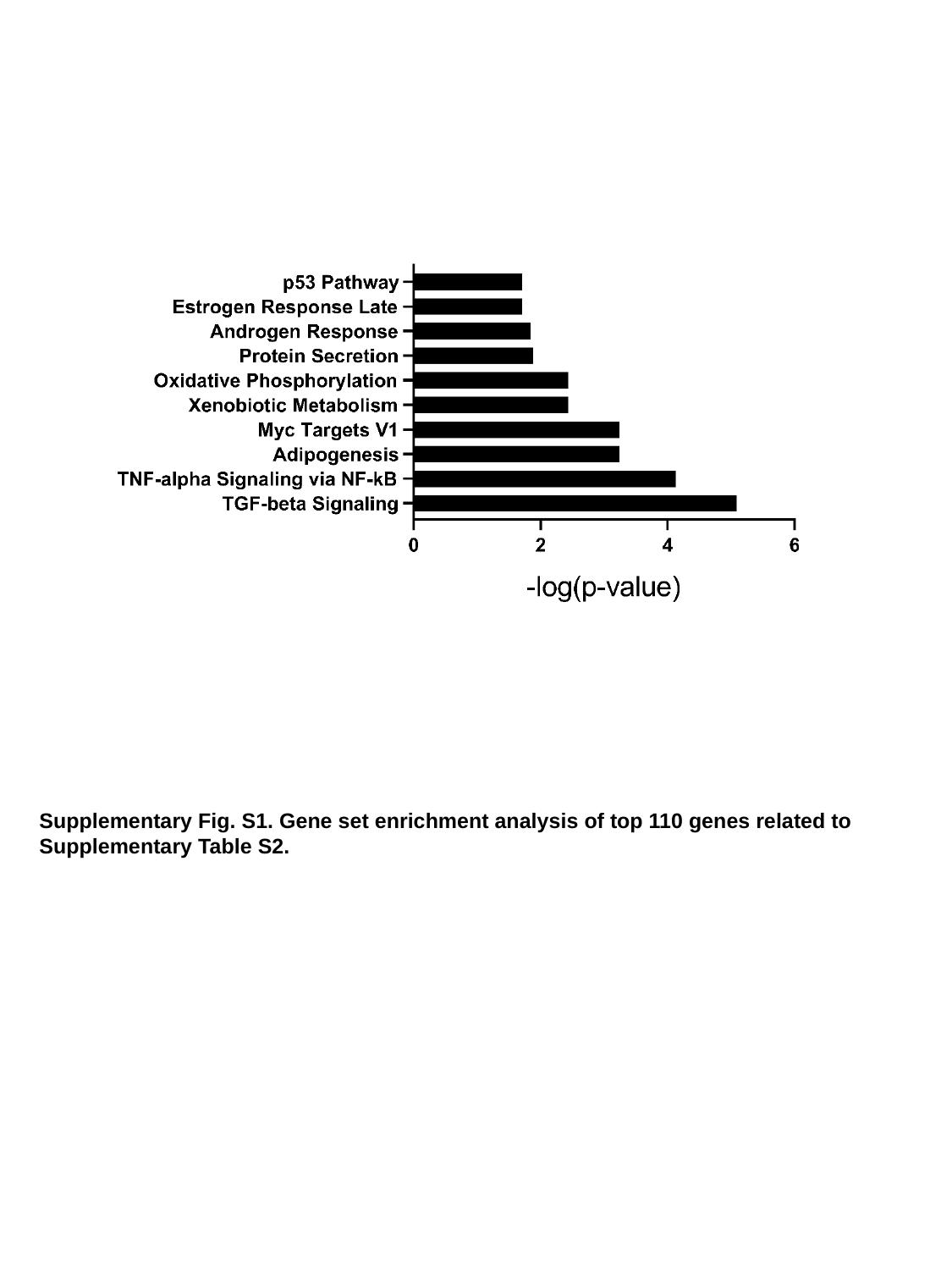

Supplementary Fig. S1. Gene set enrichment analysis of top 110 genes related to Supplementary Table S2.

## Slide 5
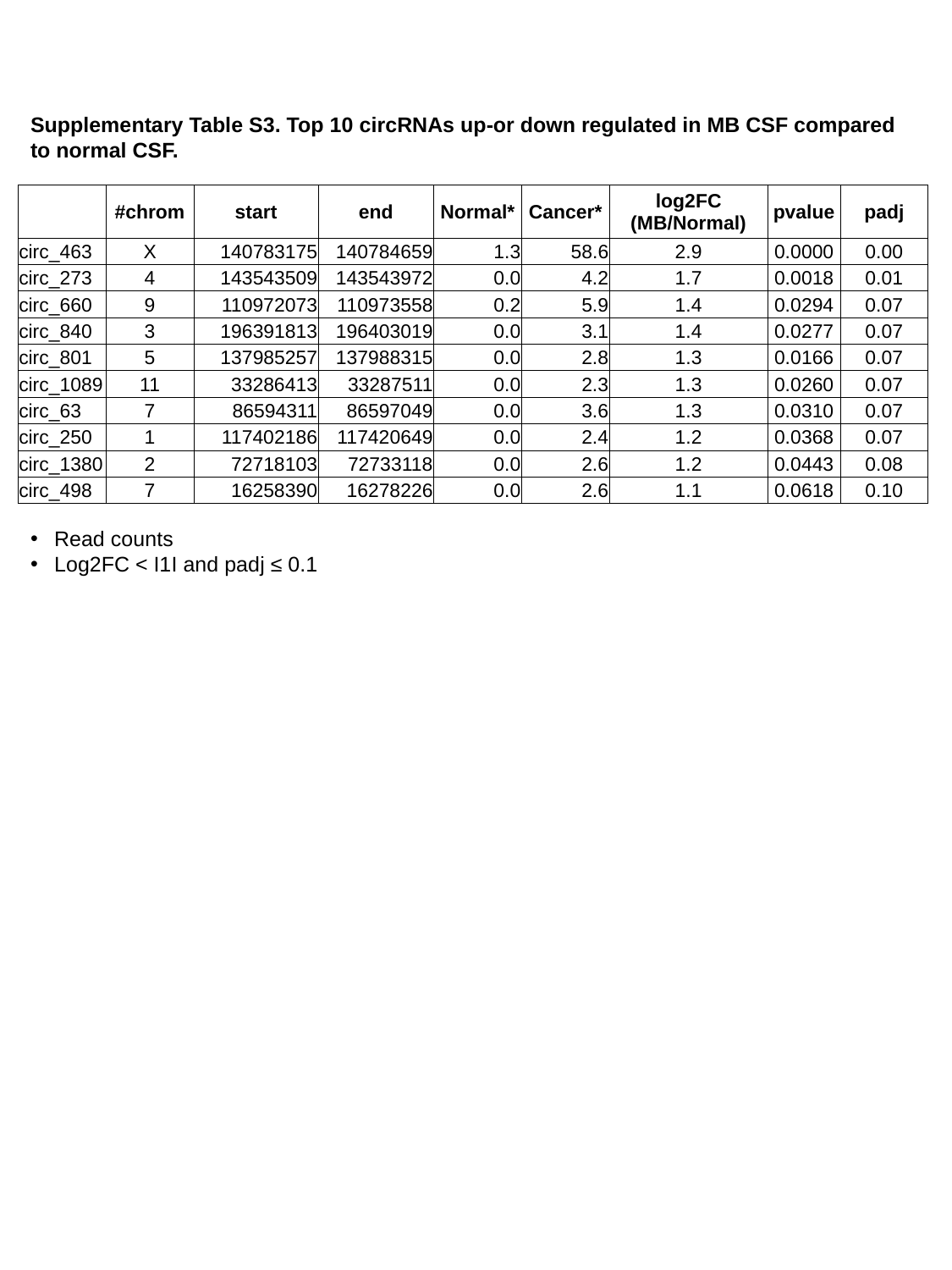

Supplementary Table S3. Top 10 circRNAs up-or down regulated in MB CSF compared to normal CSF.
| | #chrom | start | end | Normal\* | Cancer\* | log2FC (MB/Normal) | pvalue | padj |
| --- | --- | --- | --- | --- | --- | --- | --- | --- |
| circ\_463 | X | 140783175 | 140784659 | 1.3 | 58.6 | 2.9 | 0.0000 | 0.00 |
| circ\_273 | 4 | 143543509 | 143543972 | 0.0 | 4.2 | 1.7 | 0.0018 | 0.01 |
| circ\_660 | 9 | 110972073 | 110973558 | 0.2 | 5.9 | 1.4 | 0.0294 | 0.07 |
| circ\_840 | 3 | 196391813 | 196403019 | 0.0 | 3.1 | 1.4 | 0.0277 | 0.07 |
| circ\_801 | 5 | 137985257 | 137988315 | 0.0 | 2.8 | 1.3 | 0.0166 | 0.07 |
| circ\_1089 | 11 | 33286413 | 33287511 | 0.0 | 2.3 | 1.3 | 0.0260 | 0.07 |
| circ\_63 | 7 | 86594311 | 86597049 | 0.0 | 3.6 | 1.3 | 0.0310 | 0.07 |
| circ\_250 | 1 | 117402186 | 117420649 | 0.0 | 2.4 | 1.2 | 0.0368 | 0.07 |
| circ\_1380 | 2 | 72718103 | 72733118 | 0.0 | 2.6 | 1.2 | 0.0443 | 0.08 |
| circ\_498 | 7 | 16258390 | 16278226 | 0.0 | 2.6 | 1.1 | 0.0618 | 0.10 |
Read counts
Log2FC < I1I and padj ≤ 0.1

## Slide 6
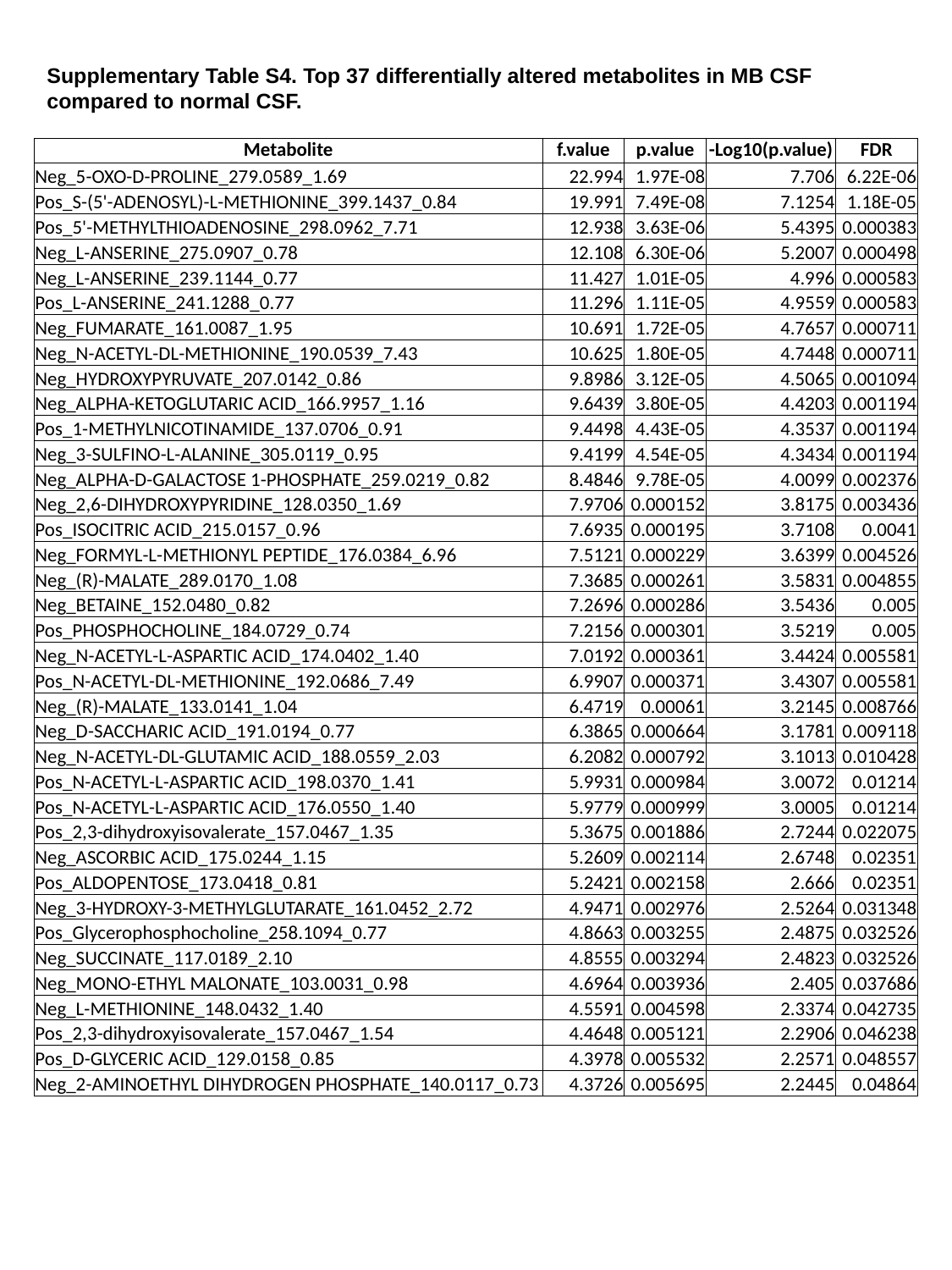

Supplementary Table S4. Top 37 differentially altered metabolites in MB CSF compared to normal CSF.
| Metabolite | f.value | p.value | -Log10(p.value) | FDR |
| --- | --- | --- | --- | --- |
| Neg\_5-OXO-D-PROLINE\_279.0589\_1.69 | 22.994 | 1.97E-08 | 7.706 | 6.22E-06 |
| Pos\_S-(5'-ADENOSYL)-L-METHIONINE\_399.1437\_0.84 | 19.991 | 7.49E-08 | 7.1254 | 1.18E-05 |
| Pos\_5'-METHYLTHIOADENOSINE\_298.0962\_7.71 | 12.938 | 3.63E-06 | 5.4395 | 0.000383 |
| Neg\_L-ANSERINE\_275.0907\_0.78 | 12.108 | 6.30E-06 | 5.2007 | 0.000498 |
| Neg\_L-ANSERINE\_239.1144\_0.77 | 11.427 | 1.01E-05 | 4.996 | 0.000583 |
| Pos\_L-ANSERINE\_241.1288\_0.77 | 11.296 | 1.11E-05 | 4.9559 | 0.000583 |
| Neg\_FUMARATE\_161.0087\_1.95 | 10.691 | 1.72E-05 | 4.7657 | 0.000711 |
| Neg\_N-ACETYL-DL-METHIONINE\_190.0539\_7.43 | 10.625 | 1.80E-05 | 4.7448 | 0.000711 |
| Neg\_HYDROXYPYRUVATE\_207.0142\_0.86 | 9.8986 | 3.12E-05 | 4.5065 | 0.001094 |
| Neg\_ALPHA-KETOGLUTARIC ACID\_166.9957\_1.16 | 9.6439 | 3.80E-05 | 4.4203 | 0.001194 |
| Pos\_1-METHYLNICOTINAMIDE\_137.0706\_0.91 | 9.4498 | 4.43E-05 | 4.3537 | 0.001194 |
| Neg\_3-SULFINO-L-ALANINE\_305.0119\_0.95 | 9.4199 | 4.54E-05 | 4.3434 | 0.001194 |
| Neg\_ALPHA-D-GALACTOSE 1-PHOSPHATE\_259.0219\_0.82 | 8.4846 | 9.78E-05 | 4.0099 | 0.002376 |
| Neg\_2,6-DIHYDROXYPYRIDINE\_128.0350\_1.69 | 7.9706 | 0.000152 | 3.8175 | 0.003436 |
| Pos\_ISOCITRIC ACID\_215.0157\_0.96 | 7.6935 | 0.000195 | 3.7108 | 0.0041 |
| Neg\_FORMYL-L-METHIONYL PEPTIDE\_176.0384\_6.96 | 7.5121 | 0.000229 | 3.6399 | 0.004526 |
| Neg\_(R)-MALATE\_289.0170\_1.08 | 7.3685 | 0.000261 | 3.5831 | 0.004855 |
| Neg\_BETAINE\_152.0480\_0.82 | 7.2696 | 0.000286 | 3.5436 | 0.005 |
| Pos\_PHOSPHOCHOLINE\_184.0729\_0.74 | 7.2156 | 0.000301 | 3.5219 | 0.005 |
| Neg\_N-ACETYL-L-ASPARTIC ACID\_174.0402\_1.40 | 7.0192 | 0.000361 | 3.4424 | 0.005581 |
| Pos\_N-ACETYL-DL-METHIONINE\_192.0686\_7.49 | 6.9907 | 0.000371 | 3.4307 | 0.005581 |
| Neg\_(R)-MALATE\_133.0141\_1.04 | 6.4719 | 0.00061 | 3.2145 | 0.008766 |
| Neg\_D-SACCHARIC ACID\_191.0194\_0.77 | 6.3865 | 0.000664 | 3.1781 | 0.009118 |
| Neg\_N-ACETYL-DL-GLUTAMIC ACID\_188.0559\_2.03 | 6.2082 | 0.000792 | 3.1013 | 0.010428 |
| Pos\_N-ACETYL-L-ASPARTIC ACID\_198.0370\_1.41 | 5.9931 | 0.000984 | 3.0072 | 0.01214 |
| Pos\_N-ACETYL-L-ASPARTIC ACID\_176.0550\_1.40 | 5.9779 | 0.000999 | 3.0005 | 0.01214 |
| Pos\_2,3-dihydroxyisovalerate\_157.0467\_1.35 | 5.3675 | 0.001886 | 2.7244 | 0.022075 |
| Neg\_ASCORBIC ACID\_175.0244\_1.15 | 5.2609 | 0.002114 | 2.6748 | 0.02351 |
| Pos\_ALDOPENTOSE\_173.0418\_0.81 | 5.2421 | 0.002158 | 2.666 | 0.02351 |
| Neg\_3-HYDROXY-3-METHYLGLUTARATE\_161.0452\_2.72 | 4.9471 | 0.002976 | 2.5264 | 0.031348 |
| Pos\_Glycerophosphocholine\_258.1094\_0.77 | 4.8663 | 0.003255 | 2.4875 | 0.032526 |
| Neg\_SUCCINATE\_117.0189\_2.10 | 4.8555 | 0.003294 | 2.4823 | 0.032526 |
| Neg\_MONO-ETHYL MALONATE\_103.0031\_0.98 | 4.6964 | 0.003936 | 2.405 | 0.037686 |
| Neg\_L-METHIONINE\_148.0432\_1.40 | 4.5591 | 0.004598 | 2.3374 | 0.042735 |
| Pos\_2,3-dihydroxyisovalerate\_157.0467\_1.54 | 4.4648 | 0.005121 | 2.2906 | 0.046238 |
| Pos\_D-GLYCERIC ACID\_129.0158\_0.85 | 4.3978 | 0.005532 | 2.2571 | 0.048557 |
| Neg\_2-AMINOETHYL DIHYDROGEN PHOSPHATE\_140.0117\_0.73 | 4.3726 | 0.005695 | 2.2445 | 0.04864 |

## Slide 7
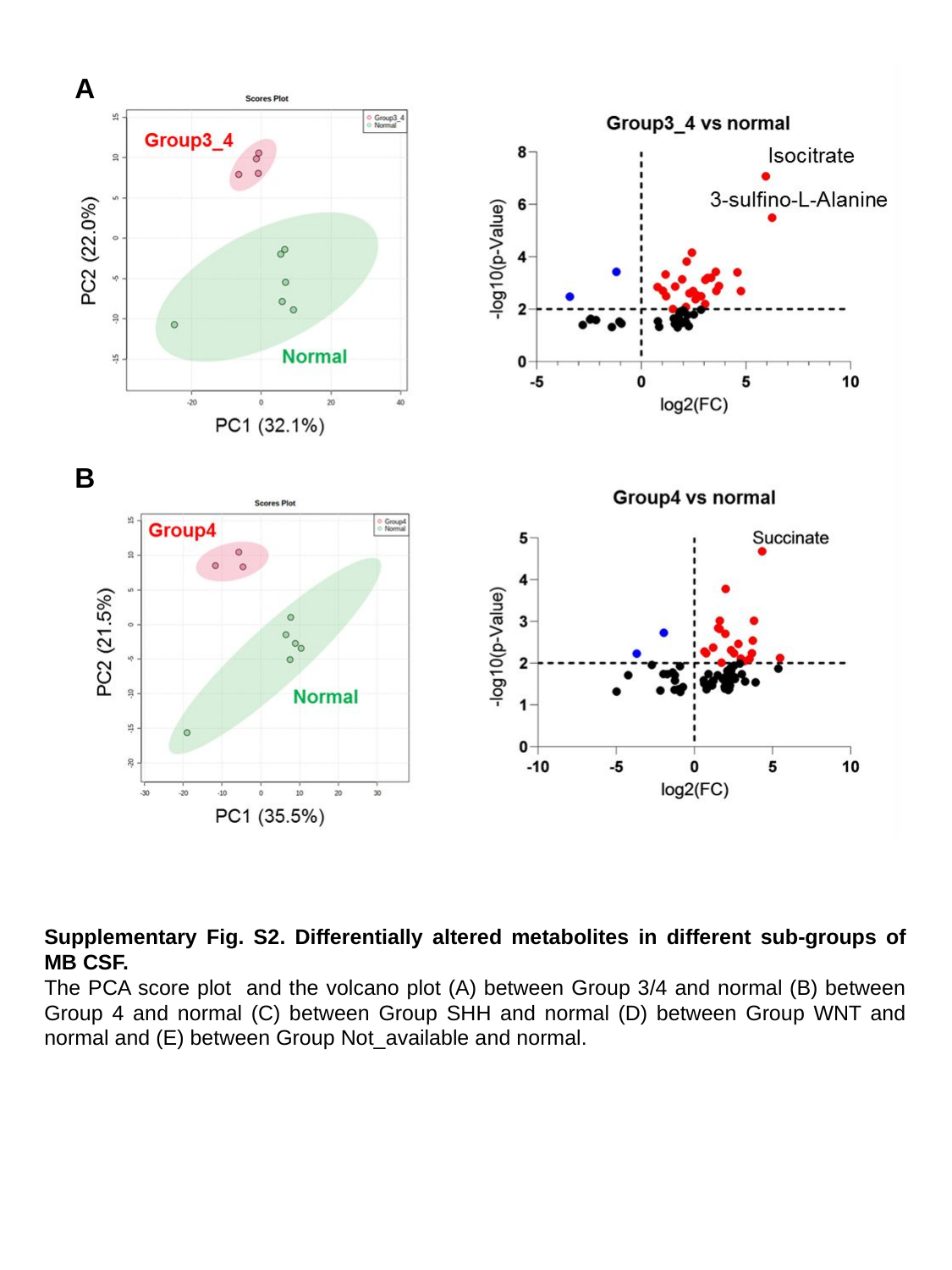

A
B
Supplementary Fig. S2. Differentially altered metabolites in different sub-groups of MB CSF.
The PCA score plot and the volcano plot (A) between Group 3/4 and normal (B) between Group 4 and normal (C) between Group SHH and normal (D) between Group WNT and normal and (E) between Group Not_available and normal.

## Slide 8
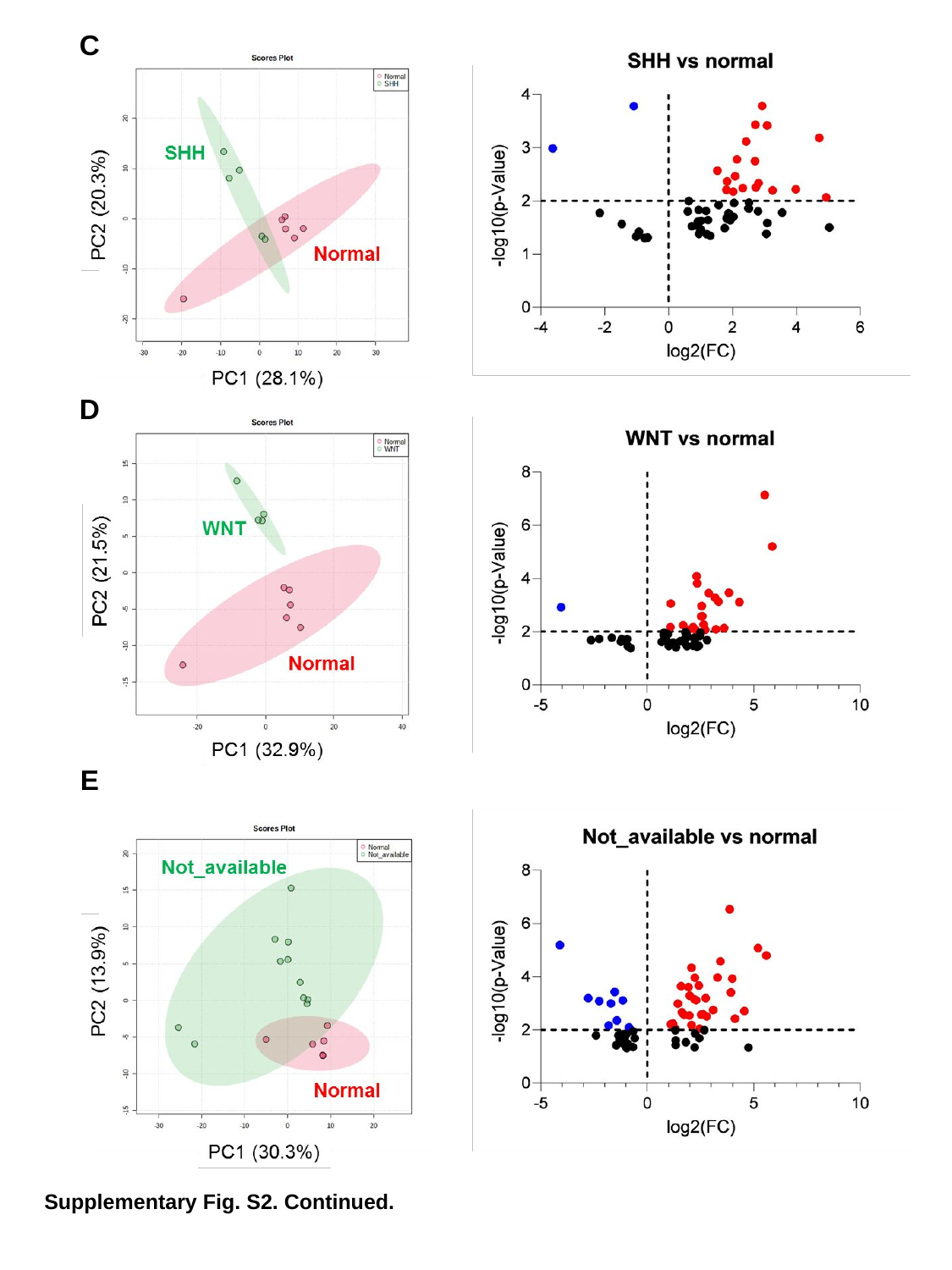

C
D
E
Supplementary Fig. S2. Continued.

## Slide 9
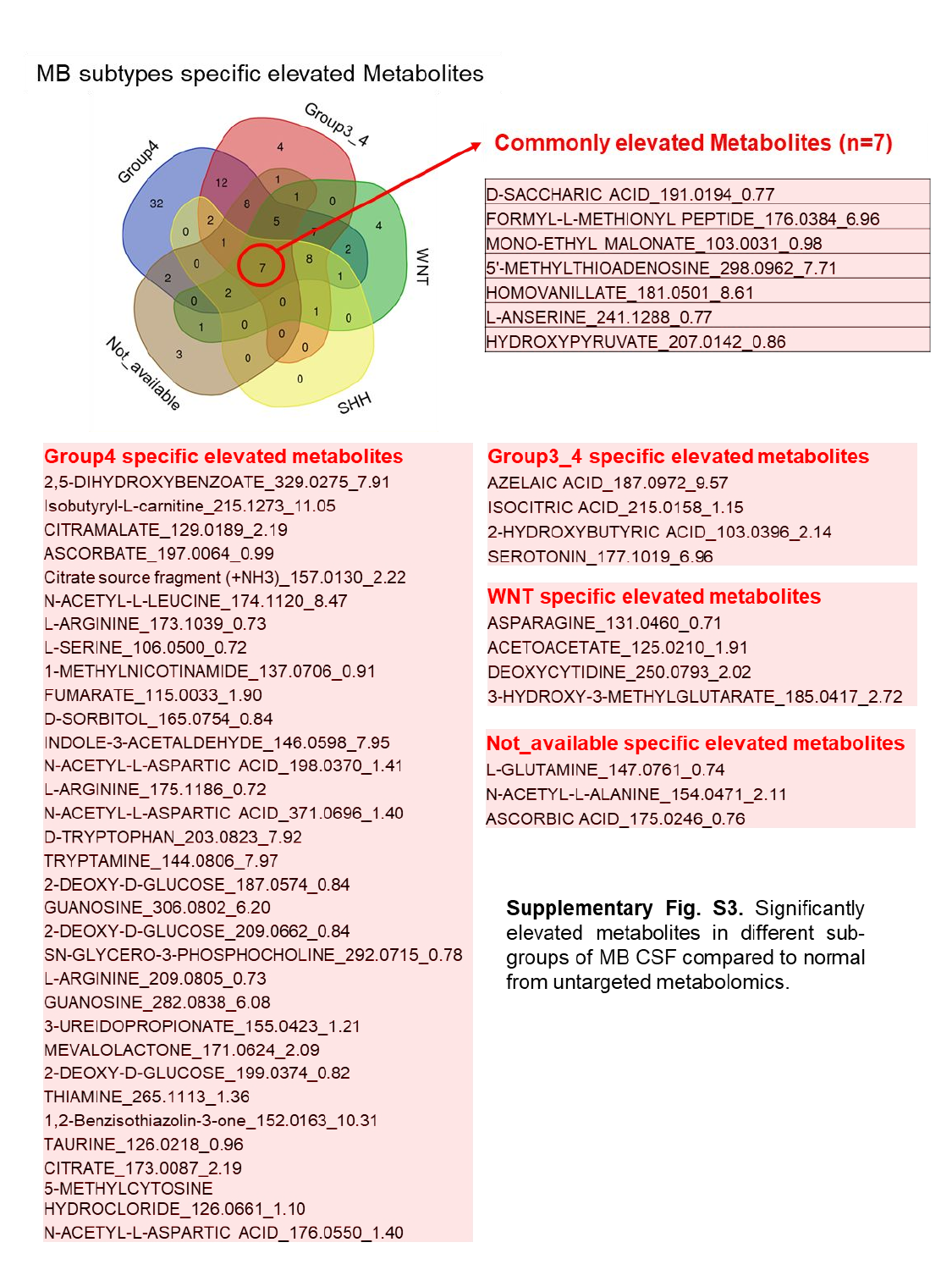

## Slide 10
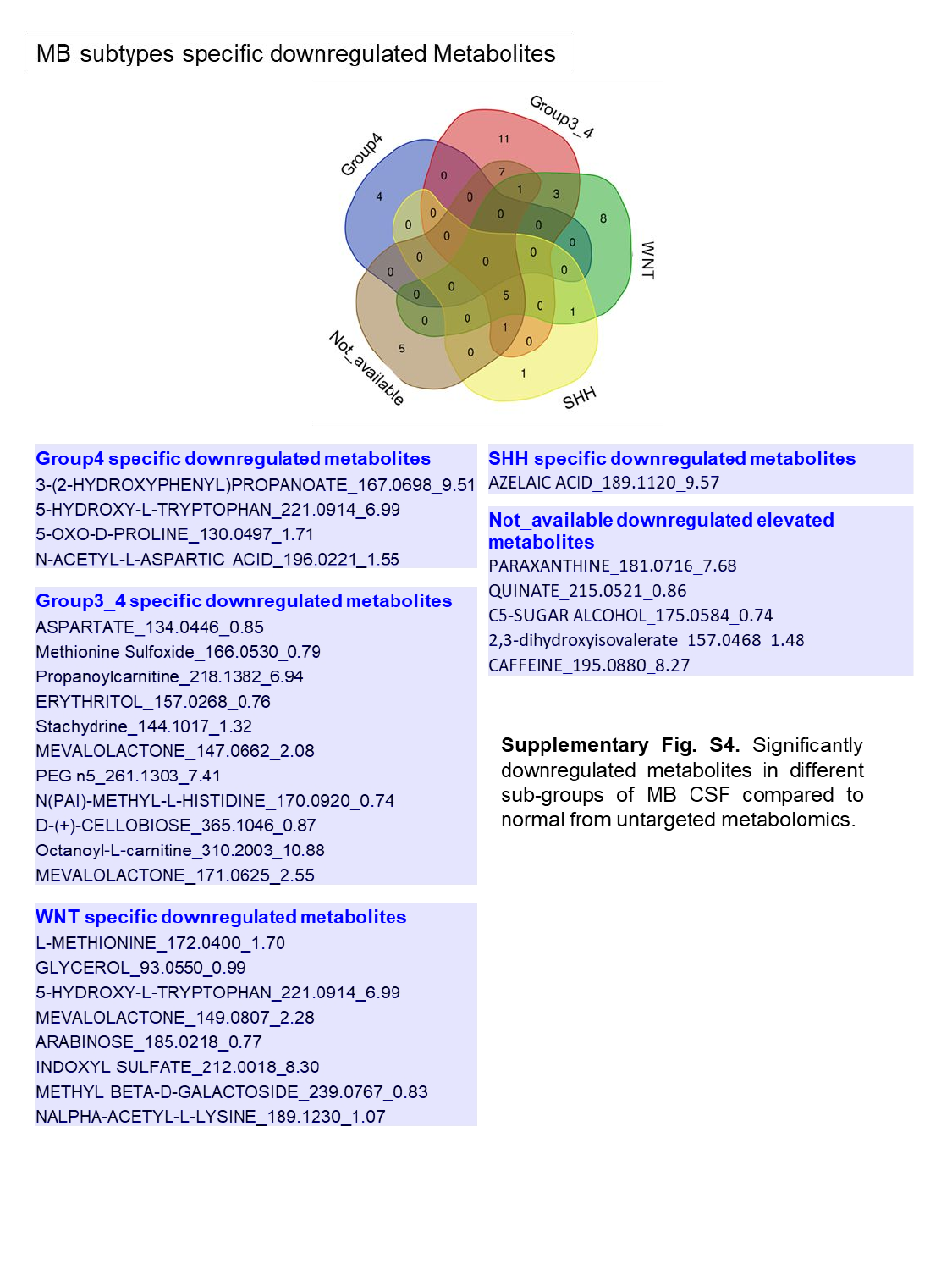

## Slide 11
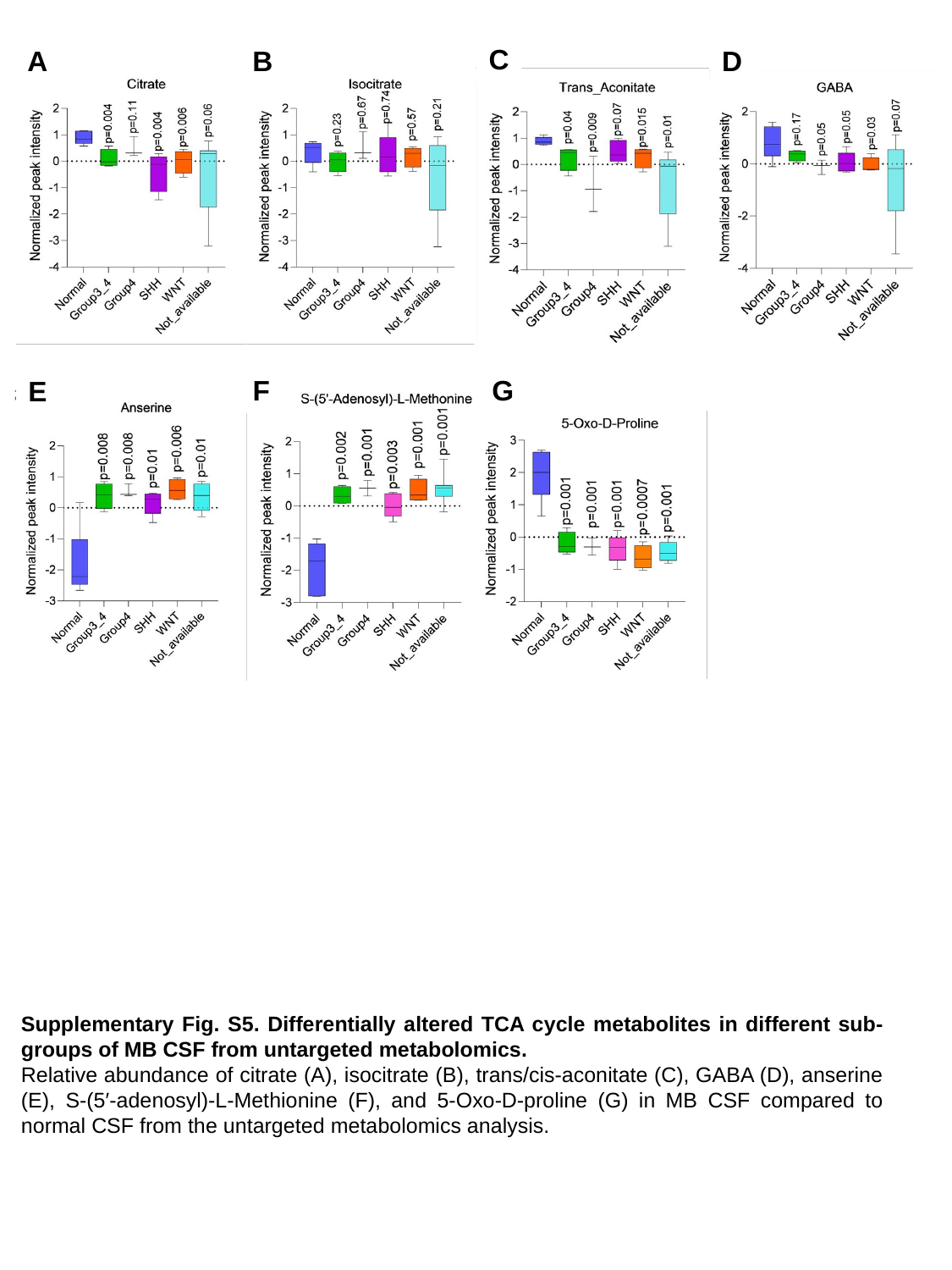

C
A
B
D
F
G
E
Supplementary Fig. S5. Differentially altered TCA cycle metabolites in different sub-groups of MB CSF from untargeted metabolomics.
Relative abundance of citrate (A), isocitrate (B), trans/cis-aconitate (C), GABA (D), anserine (E), S-(5′-adenosyl)-L-Methionine (F), and 5-Oxo-D-proline (G) in MB CSF compared to normal CSF from the untargeted metabolomics analysis.

## Slide 12
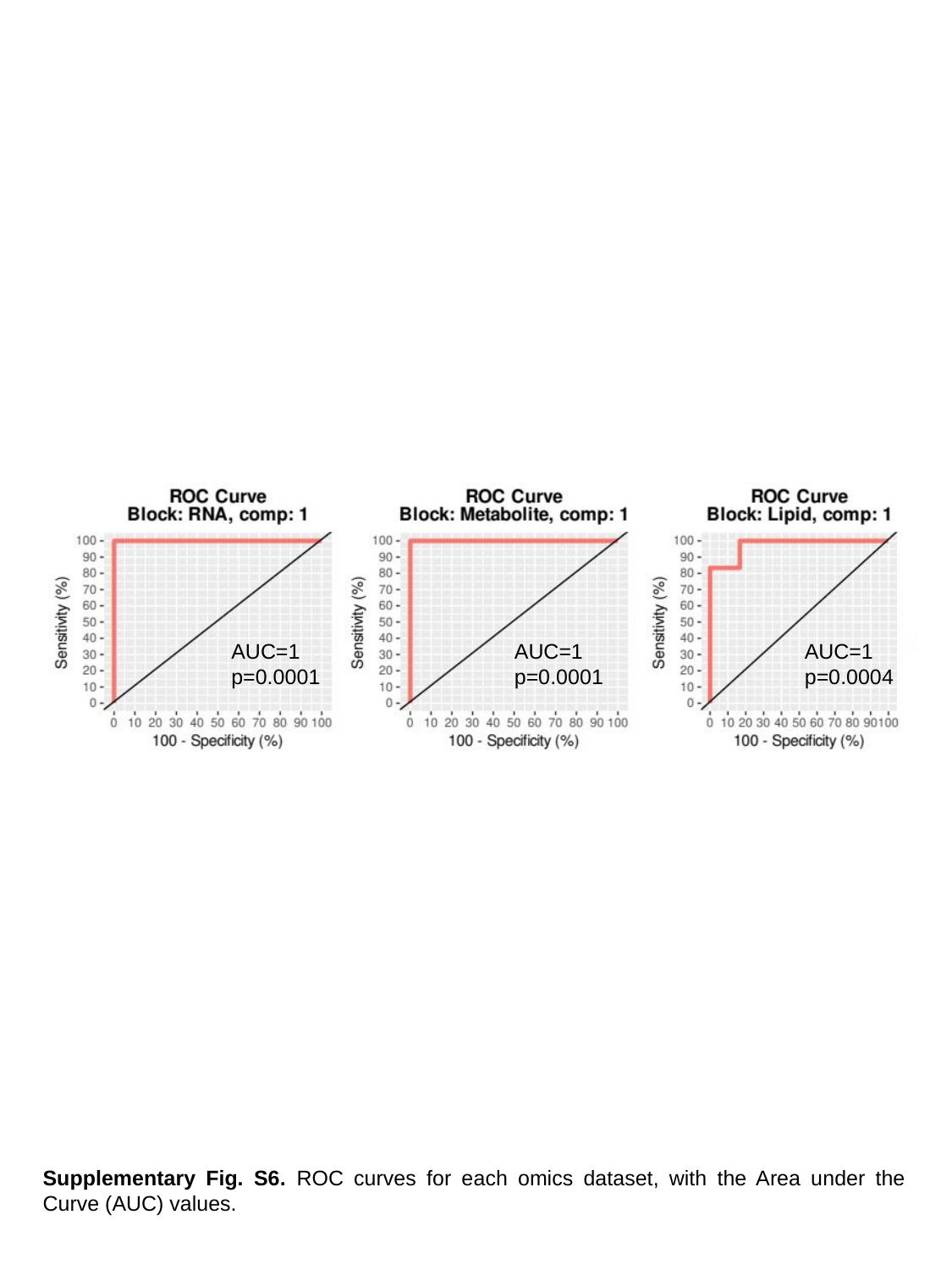

AUC=1
p=0.0004
AUC=1
p=0.0001
AUC=1
p=0.0001
Supplementary Fig. S6. ROC curves for each omics dataset, with the Area under the Curve (AUC) values.

## Slide 13
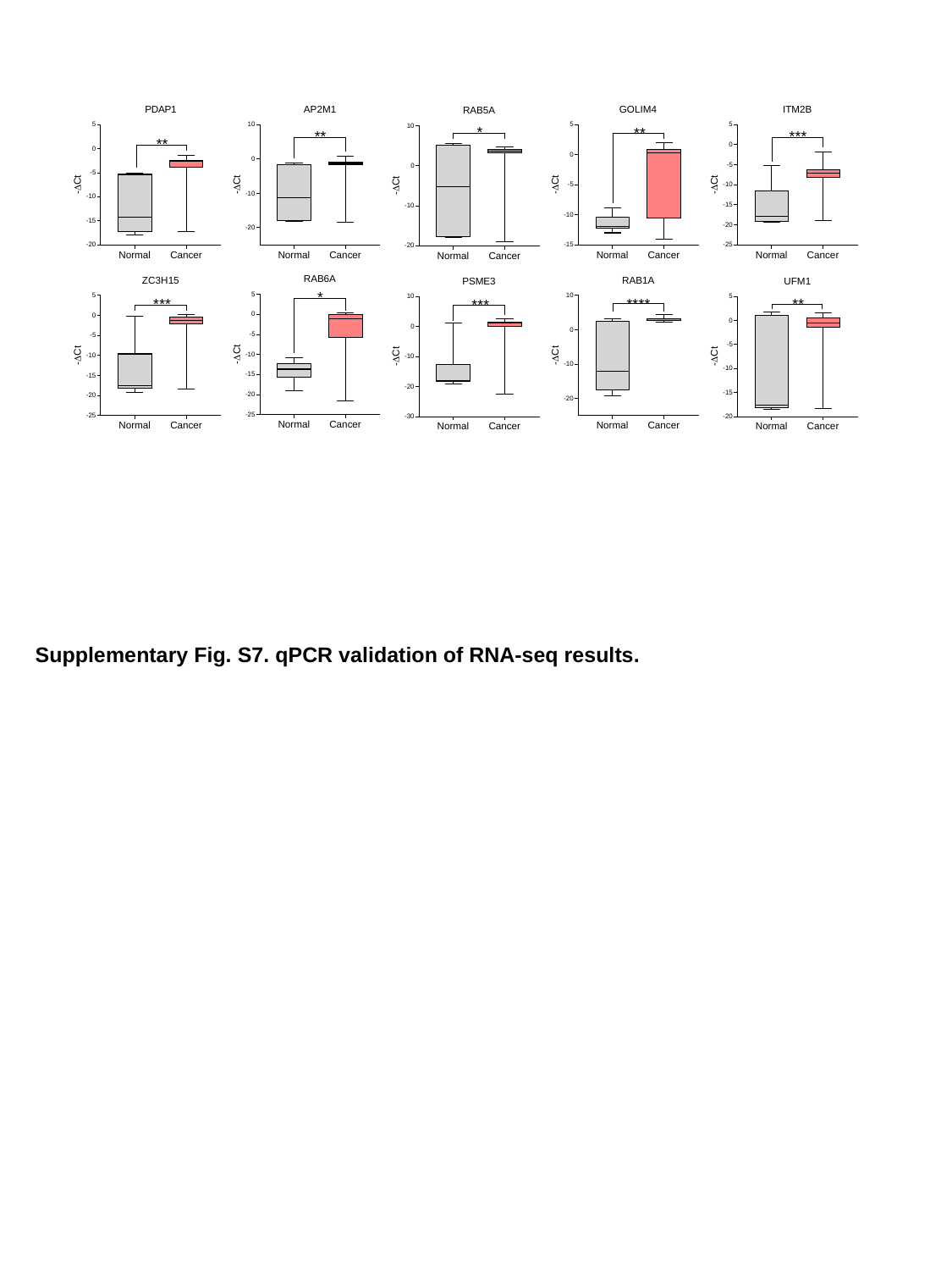

Supplementary Fig. S7. qPCR validation of RNA-seq results.

## Slide 14
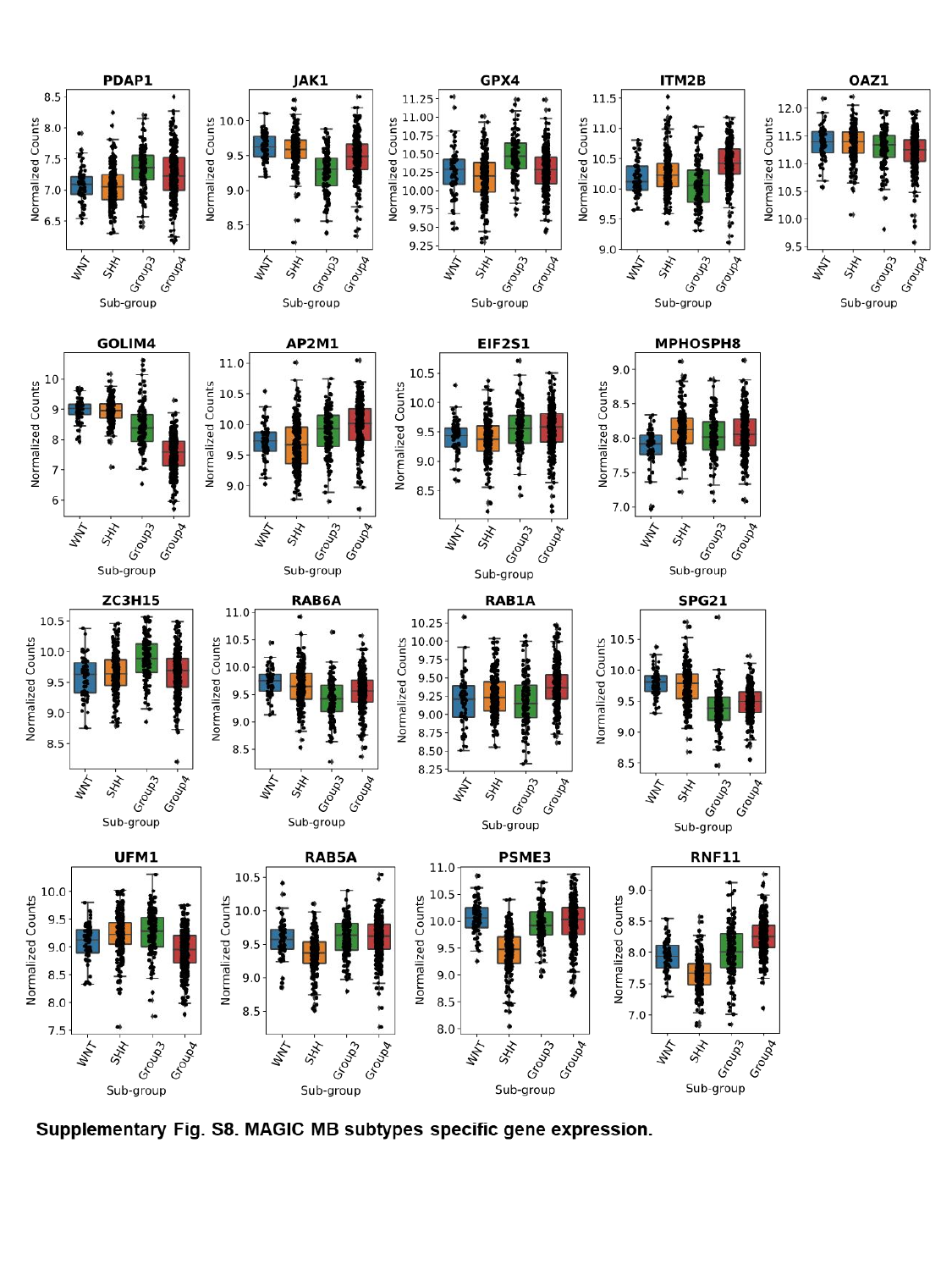

## Slide 15
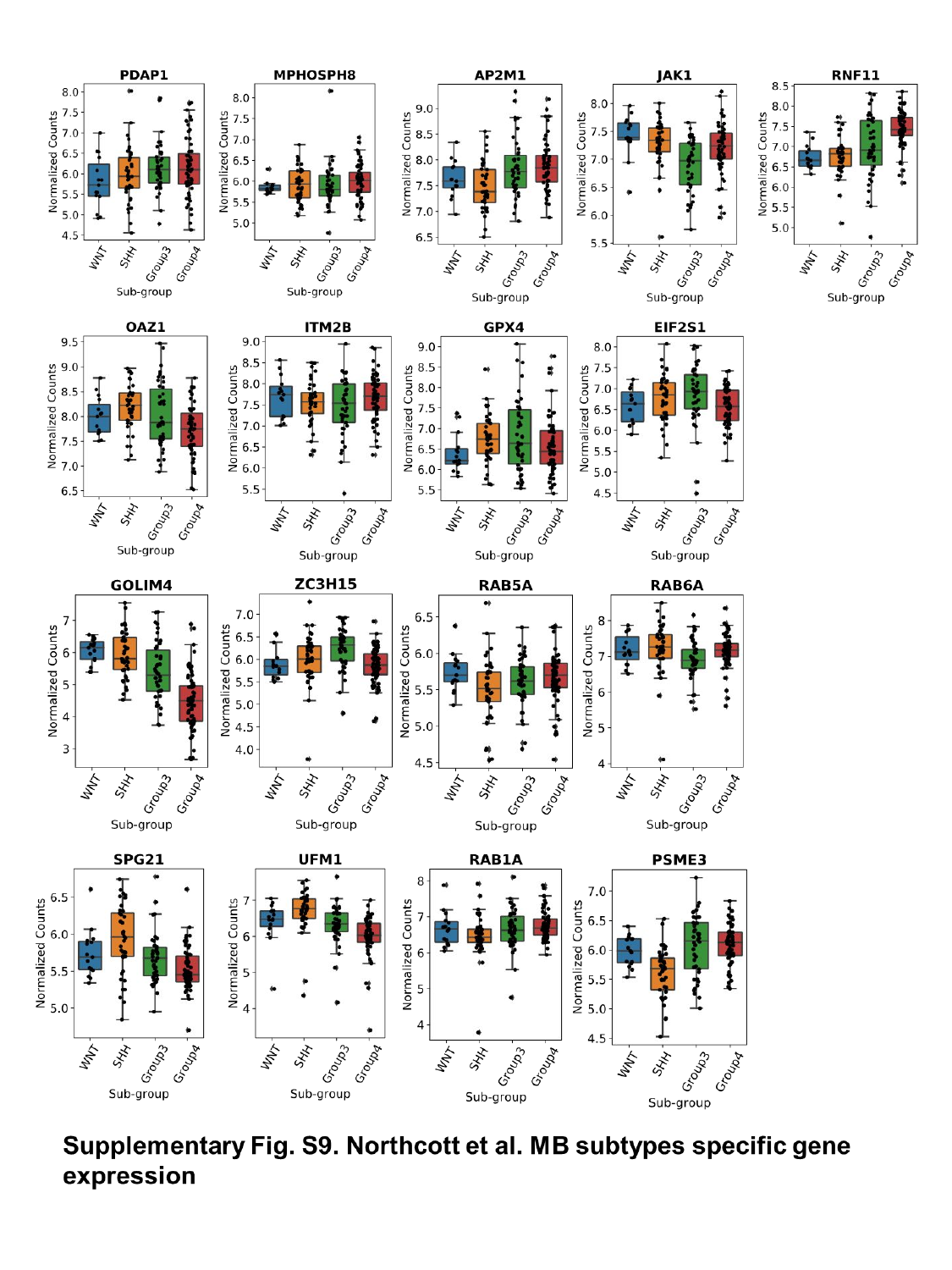

Supplement: Supplementary file 4 — Additional file 4.: Supplementary tables and figures. [file 40478_2022_1326_MOESM4_ESM.pptx]
